# RNA Epigenetics in Cancer: Current Knowledge and Therapeutic Implications

**DOI:** 10.1002/mco2.70322

**Published:** 2025-08-03

**Authors:** Shanhe Huang, Zonglin Li, Weilong Lin, Ruihui Xie, Hai Huang

**Affiliations:** ^1^ Department of Urology Sun Yat‐sen Memorial Hospital, Sun Yat‐sen University Guangzhou China; ^2^ Guangdong Provincial Key Laboratory of Malignant Tumor Epigenetics and Gene Regulation Sun Yat‐Sen Memorial Hospital, Sun Yat‐Sen University Guangzhou China; ^3^ Guangdong Provincial Clinical Research Center for Urological Diseases Sun Yat‐Sen Memorial Hospital, Sun Yat‐Sen University Guangzhou China; ^4^ Department of Urology The Sixth Affiliated Hospital of Guangzhou Medical University, Qingyuan People's Hospital Qingyuan China

**Keywords:** RNA epigenetics, RNA metabolism, pan‐cancer, targeted therapy

## Abstract

RNA epigenetics, also referred to as epitranscriptomics, has emerged as a critical regulatory layer in cancer biology, extending beyond the scope of traditional DNA and histone modifications. It encompasses a series of dynamic posttranscriptional processes—including RNA biosynthesis, splicing, transport, stability, degradation, translation, and chemical modifications—orchestrated by RNA‐binding proteins (RBPs) and noncoding RNAs (ncRNAs). Collectively, these mechanisms influence mRNA fate, shape transcriptional output, and reprogram the tumor microenvironment. Importantly, both coding RNA and ncRNA are themselves subjected to epigenetic regulation, forming intricate feedback loops that contribute to oncogenesis, immune evasion, metastasis, and therapeutic resistance. In this review, we systematically synthesize the current understanding of RNA metabolism and RNA epigenetic modifications during tumor progression, with a particular focus on the roles of RBPs and RNA modifications. Furthermore, we highlight recent advances in RNA‐based therapeutic strategies, including mRNA vaccines, antisense oligonucleotides, siRNAs, and circRNA scaffolds. These innovative approaches offer promising avenues for targeting transcriptionally active yet genomically “undruggable” cancer drivers. Together, our synthesis provides a comprehensive framework for understanding RNA epigenetics in tumor biology and lays the groundwork for precision oncology guided by transcriptome plasticity.

## Graphical abstract

RNA epigenetics regulates cancer through RNA modifications, splicing, transport, and translation. Key components include m⁶A writers, splicing factors, RBPs, and nuclear export machinery. Aberrant RNA metabolism drives tumor progression and immune evasion. Emerging therapies target RNA modifiers (e.g., METTL3 inhibitors, circRNA sponges), offering new opportunities for RNA‐based precision oncology.

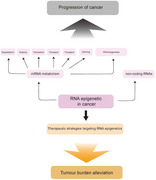

## 1 Introduction

As a branch of epigenetics, RNA epigenetics, also known as epitranscriptomics, encompasses the study of regulatory mechanisms governing coding and ncRNAs, which critically regulate expression levels [1]. While epigenetic research has traditionally focused on DNA and histone modifications, RNA plays a central role in gene expression through processes such as biosynthesis, splicing, transport, stability control, degradation, translation, and chemical modification—collectively termed RNA metabolism [2]. Based on the intermediate position of RNA, RNA metabolism connects epigenetics such as DNA modification, proteomic modification, and chromatin remodeling, which refers to regulating gene expression without altering the original DNA sequence, and further inducing transcription of oncogenes, splicing events leading to malignant phenotypes, and expression of oncogenes, which can induce abnormal cellular behavior and ultimately promote the development of cancer [3, 4, 5]. The role of the immune system in tumor biology is also widely acknowledged, and RNA epigenetics remodels the tumor immune microenvironment, promoting a protumorigenic landscape [6, 7]. Based on this, defining and deeply understanding the RNA metabolism process during tumor progression will help to comprehensively grasp tumor cells and tumor microenvironment (TME) at the molecular level, and further improve the diagnosis and treatment of various tumors.

During RNA epigenetic, RNA‐binding proteins (RBPs) and ncRNAs directly participate in RNA regulation [8, 9], making them attractive targets for therapeutic intervention. In fact, recent advances in technologies such as high‐throughput RNA analysis have led to an exponential expansion in our understanding of the epigenetic regulation of RNA metabolism [10], with promising progress made in translating RNA epigenetics‐based therapeutic strategies. For instance, targeting RNA modifications to enhance the stability and translational efficiency of exogenous RNAs underscores the potential of RNA vaccines as an emerging cancer therapeutic strategy [11]. At the same time, directly targeting core enzymes involved in RNA epigenetic regulation or their key upstream and downstream molecules can inhibit cancer cell biological functions to exert therapeutic effects, circumventing the genomic alterations associated with traditional gene therapy [12].

Here, we reviewed the RNA epigenetic processes underlying tumor progression, encompassing the roles and impacts of RNA metabolism and ncRNAs on both tumor cells and immune cell populations. We further elaborate on the advantages and feasibility of RNA‐based therapeutic strategies, offering novel perspectives and recommendations for future developments in RNA therapy.

## 2 Epigenetics of RNA Metabolism in Cancer Progression

RNA metabolism comprises transcription, splicing, transport, stabilization, degradation, translation, and chemical modification [2]. Collectively, these processes regulate gene expression through control of mRNA abundance, structural diversity, and protein localization. During tumorigenesis, aberrant RNA epigenetics drives oncogene and tumor suppressor dysregulation through mechanisms independent of genomic alterations [13, 14]. In cancer, alternative splicing (AS) generates distinct transcript isoforms that promote tumor progression [15]. In addition to regulating tumor‐intrinsic pathways, RNA epigenetics also plays a key role in shaping the tumor immune microenvironment by modulating the function and differentiation of immune cells [6]. To maintain the homeostasis of the RNA epigenetic landscape, cells employ multilayered regulatory mechanisms across all RNA metabolic stages. RBPs, via their specialized RNA recognition motifs, directly regulate RNA metabolism and epigenetic states [16, 17]. Among these regulatory mechanisms, posttranscriptional RNA modifications—including N6‐methyladenosine (m^6^A), 5‐methylcytosine (m^5^C), and others—have emerged as critical regulators of RNA fate. These modifications are introduced, removed, and interpreted by specific enzymes, collectively termed writers, erasers, and readers. In both tumor and immune cells, perturbations in one or more components of the RNA metabolic machinery can induce widespread transcriptomic alterations [14]. These aberrations subsequently promote oncogenic transformation, immune evasion, and therapeutic resistance [13, 18]. Thus, elucidating RNA metabolism and its dysregulation is essential for identifying novel therapeutic targets.

### 2.1 RNA Biosynthesis is the Foundation of RNA Metabolism Process

**FIGURE 1:**
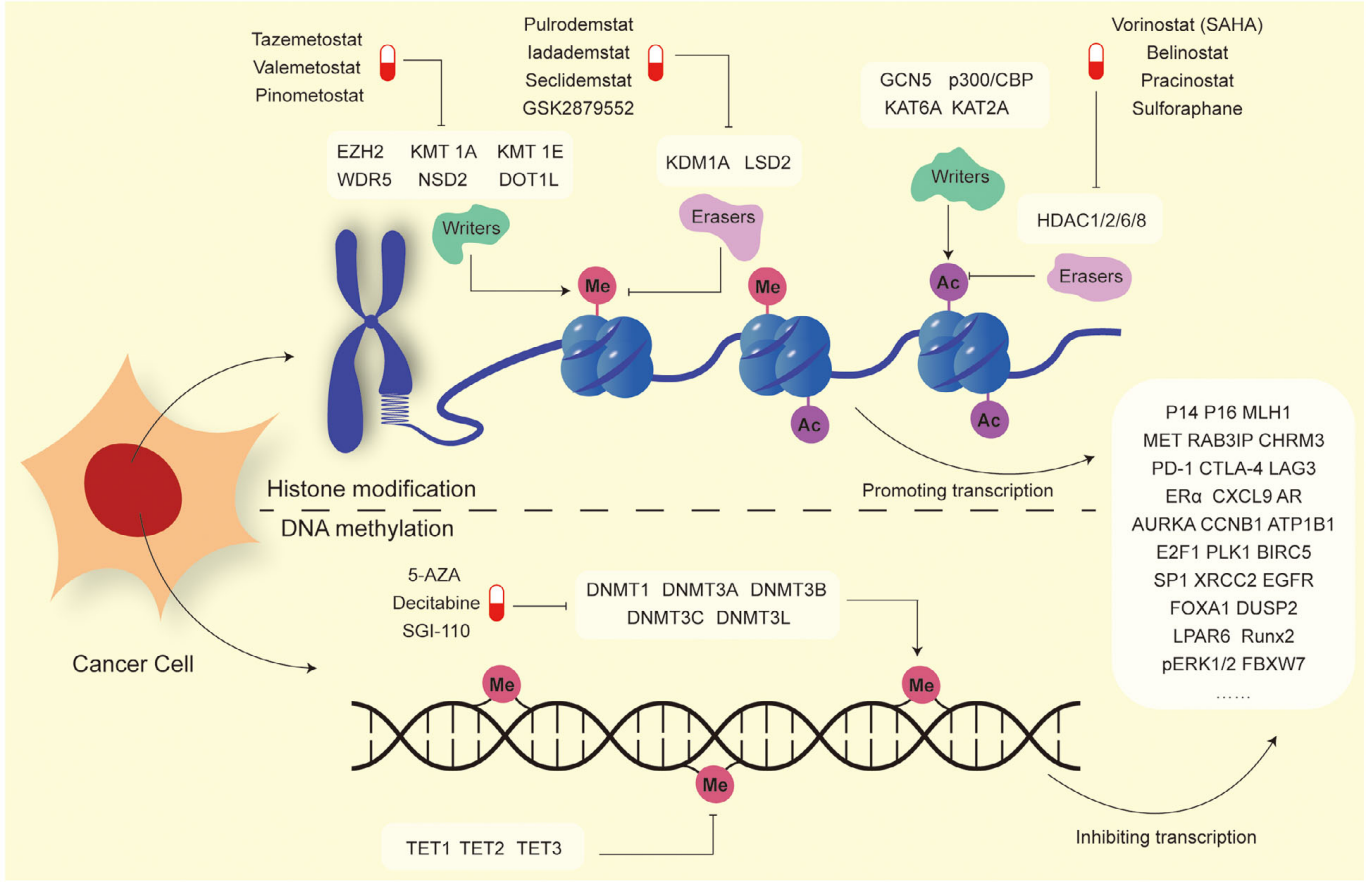
RNA biogenesis during the cancer progression. The figure illustrates histone modifications and DNA methylation involved in cancer progression. The upper part depicts histone modifications, where histones exist as octamers composed of core histone proteins (H2A, H2B, H3, and H4). The N‐terminal tails of these histones can be chemically modified. This section highlights histone methylation and acetylation‐related enzymes implicated in tumor development, along with representative therapeutic agents targeting these modifications. The lower part of the figure focuses on DNA methylation in cancer. DNA methylation typically represses gene expression at methylated loci. DNA methyltransferases (DNMTs) function as “writers” that catalyze the addition of methyl groups, while the ten‐eleven translocation (TET) family of enzymes act as “erasers” that remove DNA methylation marks. Together, histone modifications and DNA methylation cooperatively regulate the expression of target genes, thereby contributing to tumor progression. The figure also lists cancer‐related genes that are known to be regulated by these epigenetic mechanisms.

RNA biogenesis initiates all RNA metabolic, with transcriptional activity directly reflecting gene expression [19, 20]. Different genes are transcribed concurrently with varying efficiencies, shaping complex expression profiles. In the nucleus, chromosomal architecture dictates transcriptional activity. Topoisomerases and helicases remodel chromatin by unwinding supercoiled DNA, displacing histones, and facilitating RNA polymerase recruitment [21]. Chromatin conformation, histone status, and DNA accessibility directly influence gene transcription. Dynamic epigenetic alterations regulate gene activation or silencing. Recent studies have suggested that normal cells can undergo malignant transformation solely through DNA methylation and histone modifications without requiring genetic mutations, highlighting the independent and pivotal role of epigenetics in tumorigenesis [22]. Aberrant DNA methylation and histone modifications reprogram malignant cell behavior and foster an immunosuppressive microenvironment, promoting tumor progression [20]. Thus, enzymes regulating DNA methylation and histone modifications are promising biomarkers and therapeutic targets (Figure 1).

#### 2.1.1 DNA Methylation During RNA Biogenesis

Cytosine methylation at the fifth carbon position (5mC) in DNA is a well‐established epigenetic marker involved in regulating gene expression [23]. Methylation‐induced alterations in chromatin structure restrict DNA accessibility, thereby suppressing gene transcription [23, 24]. CpG islands—regions enriched in CpG dinucleotides, typically located in gene promoters or exons—are commonly modified by 5mC [25], though their methylation levels remain low in normal cells [26]. In contrast, cancer cells exhibit widespread DNA hypermethylation, particularly in tumor suppressor genes. During the early stages of tumorigenesis, hypermethylation of genes involved in DNA repair, cell cycle control, and apoptosis is frequently observed, and this has been proposed as a biomarker for early cancer detection [27]. For example, in pediatric T‐cell acute lymphoblastic leukemia, CpG island methylation‐based stratification has been shown to overcome the limitations of classical biomarkers, offering predictive value for patient prognosis and guiding subsequent therapeutic decisions [28].

DNA methylation is a dynamic and reversible epigenetic modification regulated by a range of functionally distinct enzymes. The DNA methyltransferase (DNMT) family—including DNMT1, DNMT3A, DNMT3B, DNMT3C, and DNMT3L—mediates the addition of methyl groups to cytosine residues [29]. These DNMTs, acting as “writers” of methylation marks, are frequently dysregulated in tumors, leading to aberrant hypermethylation and silencing of tumor suppressor genes. The ten‐eleven translocation (TET) family of enzymes, consisting of TET1, TET2, and TET3, are the only known “erasers” of DNA methylation [30], catalyzing the oxidation of 5mC to 5‐hydroxymethylcytosine (5hmC) and other intermediates. Additionally, DNA methylation is interpreted by “reader” proteins, such as those in the methyl‐CpG‐binding domain (MBD) family [31]. For instance, MECP2, a member of the MBD protein family, binds to methylated CpG sites and recruits corepressor complexes, thereby inducing repressive chromatin states and contributing to tumor progression [32, 33].

In tumors, DNA hypermethylation frequently occurs in tumor suppressor genes and mismatch repair genes [24], leading to their transcriptional silencing. DNA methylation drives chromatin condensation into transcriptionally inactive heterochromatin, thereby preventing transcription factors from binding to gene promoters and inhibiting gene expression [25]. For instance, hypermethylation of P16 and P14 is commonly observed in various cancers [34, 35, 36, 37], contributing to the loss of cell cycle regulation and evasion of apoptosis, respectively. Methylation of the tumor suppressor gene RASSF1A [38, 39], one of the most frequently altered genes in cancer, one of the most frequently altered genes in cancer, promotes tumor proliferation, and survival via the Ras/Raf/MAP kinase‐ERK kinase (MEK)/extracellular‐signal‐regulated kinase (ERK) signaling pathway [40]. Additionally, MLH1 hypermethylation is associated with colorectal and lung cancer vascular invasion, as well as tumor growth and metastasis in endometrial cancer [41, 42]. Promoter methylation of O6‐methylguanine–DNMT (MGMT) [43], a DNA repair enzyme responsible for preventing mutagenesis and cell cycle arrest, is frequently observed in glioblastomas [44], gastric [45], breast, and rectal cancers [46] and is associated with poor prognosis. Recent studies in acute myeloid leukemia (AML) have revealed that epigenetic silencing of ATP1B1 through promoter‐region DNA methylation and histone modification leads to instability of the Na/K‐ATPase pump, highlighting this pathway as a potential therapeutic target [47].

Global DNA hypomethylation is another hallmark of cancer cells [48], contributing to chromosomal instability and oncogene activation [49], thereby inducing aggressive malignant phenotypes. Transposable elements [49, 50], particularly LINE‐1 sequences, are known to be hypomethylated in tumors and may act as alternative promoters, facilitating aberrant gene activation. Hypomethylation of LINE‐1 elements has been identified within intronic regions of proto‐oncogenes [51, 52], thereby enhancing their transcriptional activity. Moreover, DNA methylation shaped by methionine metabolism has been implicated in cancer cachexia, where the methionine/SAM–DNMT3A/DNA hypomethylation–Ddit4/REDD1 axis exacerbates muscle wasting, underscoring the clinical relevance of nutritional regulation in cancer patients [53]. Additionally, DNA hypomethylation has been implicated in shaping immunosuppressive TME [24]. Unlike adjacent normal tissues, where CD8+ T cells exhibit hypermethylation of immune checkpoint genes, tumor‐infiltrating CD8+ T cells often show hypomethylation of these genes, leading to impaired immune responses [54]. Similarly, low methylation levels of another immune checkpoint gene, LAG3, have been reported in several cancer types [55].

Given the aberrant methylation landscape in cancer genomes, targeting DNA methylation via inhibitors has emerged as a promising therapeutic strategy. DNMT inhibitors (DNMTi) [24, 56], such as nucleoside analogs including 5‐azacytidine (5‐AZA), 5‐aza‐2′‐deoxycytidine (decitabine), and SGI‐110 (guadecitabine), have demonstrated clinical efficacy in hematologic malignancies, including myelodysplastic syndrome (MDS), AML, and chronic myelomonocytic leukemia [57, 58, 59]. In solid tumors, DNMTi are also yielding encouraging results in clinical trials [60]. In addition, low‐dose decitabine has been shown to reverse the immunosuppressive microenvironment in triple‐negative breast cancer (TNBC) mouse models by enhancing the infiltration, function, and memory of tumor‐infiltrating lymphocytes, further supporting its potential for immune modulation [61]. TET inhibitors, which directly block the enzymatic activity of TET1/2, hold therapeutic potential in subsets of hematologic cancers, though clinical studies remain limited [62]. Furthermore, TET inhibition may aid in reversing the immunosuppressive TME by restoring CD8+ T cell cytotoxicity. Combination therapies involving DNMTi [60 ^,^ 63 ^,^ 64]—such as DNMTi combined with chemotherapy, immune checkpoint inhibitors, or histone deacetylase (HDAC) inhibitors (HDACi)—have exhibited synergistic effects in overcoming drug resistance and enhancing antitumor efficacy, representing a promising avenue for future clinical applications.

#### 2.1.2 Histone Modification During RNA Biogenesis

In highly condensed chromatin, DNA is tightly wrapped around histone octamers, forming nucleosomes. Each histone protein has an N‐terminal “tail” extending outward from the DNA–histone complex, which serves as a key site for posttranslational modifications [65]. There are various types of histone modifications, including acetylation, methylation, phosphorylation, ubiquitination, and sumoylation. These modifications vary in chemical groups, residues, positions, and levels. Collectively, they modulate chromatin structure and alter the recruitment of chromatin‐associated proteins, thereby either promoting or inhibiting gene transcription [66, 67]. In cancer, aberrant histone modifications can activate oncogenes or silence tumor suppressor genes without altering the underlying DNA sequence, contributing to tumorigenesis.

*Methylation*: Histone methylation involves the transfer of one or more methyl groups from S‐adenosylmethionine to lysine or arginine residues of histone proteins, catalyzed by methyltransferase [68]. Certain types of methylation, such as H3K4me1/2/3, H3K36me1/2/3, and H3K79me1/2/3, have been demonstrated to repress gene transcription, whereas H3K9me1/2/3 and H3K27me3 methylation promote the expression of target genes [67].

EZH2, a key histone methyltransferase (HMT) and the catalytic subunit of the polycomb repressive complex 2, mediates H3K27 me3 and is frequently overexpressed in various cancers [69]. In pancreatic cancer cells, EZH2 regulates epithelial–mesenchymal transition (EMT) markers by increasing mesenchymal markers such as vimentin and downregulates epithelial markers including E‐cadherin, thereby promoting EMT [70]. In non‐small cell lung cancer (NSCLC), EZH2 activates the PI3K/AKT pathway, contributing to acquired resistance to gefitinib [71]. Additionally, EZH2 regulates tamoxifen resistance in breast cancer through the EZH2–ERα–GREB1 transcriptional axis [72]. Within the TME, EZH2 suppresses the production of the Th1‐type chemokine CXCL9 [73], reducing CD8+ T cell infiltration. Beyond its methyltransferase activity, EZH2 can directly bind to the androgen receptor (AR) promoter, activating gene transcription and contributing to prostate cancer (PCa) progression [74, 75].

DOT1L, the only known H3K79 methyltransferase, catalyzes methylation associated with active transcription [76]. Aberrant DOT1L activity has been implicated in mixed‐lineage leukemia (MLL)‐rearranged leukemias, and AR‐positive PCa [77, 78]. Other HMTs, such as KMT1A and KMT1E, have been reported to be upregulated in cancer and linked to tumor invasion and proliferation [79, 80, 81]. WDR5, a component of the SET1/MLL HMT complex that catalyzes H3K4me3, regulates genes involved in cell cycle, apoptosis inhibition, DNA repair, and immune regulation—including AURKA, CCNB1, E2F1, PLK1, BIRC5, XRCC2, and PD‐L1 [82]. NSD2, a member of the nuclear receptor‐binding SET domain (NSD) family, catalyzes H3K36 dimethylation, is elevated in various tumor models, and thus may serve as a promising therapeutic target [83, 84].

Conversely, histone demethylases such as LSD1 (KDM1A) and LSD2 are overexpressed in several cancers [85, 86, 87], including AML, small cell lung cancer (SCLC), breast cancer, and colorectal cancer (CRC). LSD1 promotes PCa cell proliferation and metastasis by demethylating lysine residues and upregulating oncogenes such as FOXA1 and LPAR6 [88, 89, 90]. LSD1 also enhances AR activity by demethylating H3K9, leading to derepression of AR target genes [91]. Meanwhile, LSD2 regulates TFPI‐2 expression through H3K4me1 demethylation, supporting survival in SCLC [92]. Other members of the JMJD–KDM family also increase chromatin accessibility through histone demethylation, facilitating tumor metastasis in various cancers [93, 94]. Under hypoxic conditions induced by dense tumor stroma, decreased KDM2A activity activates the MAPK pathway, driving sustained EMT in pancreatic ductal adenocarcinoma (PDAC) [95]. An imbalance between histone methylation and demethylation contributes to genomic instability, tumor growth, metastasis, and drug resistance.

Targeting histone methylation and demethylation presents a promising strategy for cancer therapy. The EZH2 inhibitor tazemetostat has been approved by the the United States Food and Drug Administration (US FDA) for treating certain lymphomas [96], demonstrating a 69% objective response rate in EZH2‐mutant patients during a phase II clinical trial [97]. Meta‐analyses indicate that tazemetostat is well tolerated, with a low incidence of severe treatment‐related adverse events [98]. Valemetostat, a dual EZH1/2 inhibitor, has shown efficacy and safety in clinical trials involving patients with relapsed or refractory peripheral T‐cell lymphoma. Pinometostat, targeting DOT1L, has exhibited moderate clinical activity in phase I/II trials for non‐Hodgkin lymphoma and T‐cell leukemia/lymphoma [99, 100, 101], warranting further investigation in phase III studies.

Moreover, research on histone demethylase inhibitors has primarily focused on LSD1, with compounds such as pulrodemstat, tranylcypromine, ORY‐1001, and others demonstrating antitumor activity in various hematologic and neuroendocrine tumors [102]. Given the role of histone methylation in modulating the TME, these therapeutic strategies may enhance immune cell function and checkpoint expression, potentially improving the efficacy of immunotherapies [6, 103]. Ongoing clinical trials are exploring the feasibility of combining these epigenetic therapies with immunotherapy.

*Acetylation*: Histone acetylation is mediated by histone acetyltransferases (HATs), which transfer acetyl groups to the lysine residues on the N‐terminal tails of histones [104]. This neutralizes the positive charge on lysine side chains, weakens the interaction between histones and the negatively charged DNA backbone, and results in a more open chromatin conformation conducive to transcriptional activation [68]. Conversely, histone deacetylation promotes chromatin condensation and transcriptional repression.

Members of the HAT family play critical roles in cancer progression, particularly in drug resistance. GCN5, the first HAT identified, regulates gene expression, cell proliferation, and metabolism [105]. In osteosarcoma, GCN5 induces H3K27 acetylation at the Runx2 promoter, upregulating Runx2 transcription and promoting pulmonary metastasis [106]. GCN5 also contributes to tamoxifen resistance in breast cancer by downregulating p53 [107], and its knockdown enhances abiraterone sensitivity in PCa cells [108]. Another key member of the HAT family is p300/CBP. Upregulation of p300 enhances pERK1/2 transcription, contributing to BRAF inhibitor resistance in melanoma [109]. Furthermore, KAT6A can promote apoptosis and sensitize ovarian cancer cells to cisplatin [110]. In B‐cell lymphomas, decreased H3K27 acetylation suppresses FBXW7 expression, activates the NOTCH signaling pathway and downstream CCL2/CSF1, and promotes tumor cell proliferation and M2 polarization of tumor‐associated macrophages (TAMs) [111].

Aberrant expression of HDACs is associated with poor prognosis and advanced disease in various solid and hematologic malignancies [112]. In nasopharyngeal carcinoma, HDAC1 maintains histone deacetylation near the DUSP2 gene [113], facilitating metastasis. During the lung metastasis process of breast cancer, HDAC8 cooperates with the SMAD3/4 complex, which suppresses TGF‐β signaling through a feedback loop [113, 114]. In NSCLC and melanoma, HDAC6 enhances the stability of EGFR and β‐tubulin, reduces apoptosis, and contributes to therapeutic resistance [115, 116]. Importantly, HDAC1/2/6‐mediated deacetylation of the transcription factor Sp1 promotes cancer stem‐like cell proliferation and temozolomide resistance in glioblastoma [117].

Therapeutic targeting of histone acetylation preceded that of DNA methylation‐based therapies. HDACis represent the primary therapeutic approach. First‐ and second‐generation HDACis, such as Vorinostat (SAHA) and Belinostat, have received US FDA approval for lymphoma treatment [118] and have been tested in clinical trials for colorectal, pancreatic, breast, and PCas [119]. Although they have shown some efficacy, their limited activity in solid tumors and associated toxicities have hindered further development. Pracinostat, a broad‐spectrum HDACi, showed tolerability in pediatric patients with refractory solid tumors, but no objective responses were observed in translocation‐associated recurrent/metastatic sarcomas [120]. Sulforaphane, a naturally occurring compound, also modulates HDAC activity and has demonstrated safety and clinical activity in phase II trials [121], warranting further investigation.

Similar to histone methylation‐targeted therapies, HDAC inhibitors can modulate the tumor immune microenvironment by upregulating PD‐L1 expression and reducing Treg populations [122], thereby enhancing antitumor immune responses and supporting their combination with immunotherapies [123, 124]. Curcumin, a HAT targeting p300/CBP, promotes p53 acetylation in vivo [125]. A Phase II clinical trial of curcumin combined with docetaxel and prednisone demonstrated good clinical efficacy and safety, with decreased PSA levels observed in 59% of patients and partial responses in 40% of evaluable cases [126].

Despite years of research on directly targeting histone modifications, no significant clinical breakthroughs have been achieved to date. Most clinical trials have been limited to a narrow range of tumor types, significantly hindering translational application. The limited cost effectiveness of these approaches remains a major barrier to further development. In general, the efficacy of monotherapies targeting histone modifications remains inferior to that of conventional radiotherapy and chemotherapy combinations. Therefore, future efforts should focus on improving RNA‐based epigenetic regulatory networks, refining preclinical models, and optimizing the physicochemical properties of candidate compounds, with the goal of enhancing the efficacy and reducing the toxicity of combination therapies in clinical settings.

### 2.2 The Stability and Degradation of RNA Maintain its Function in Terms of Quantity

**FIGURE 2:**
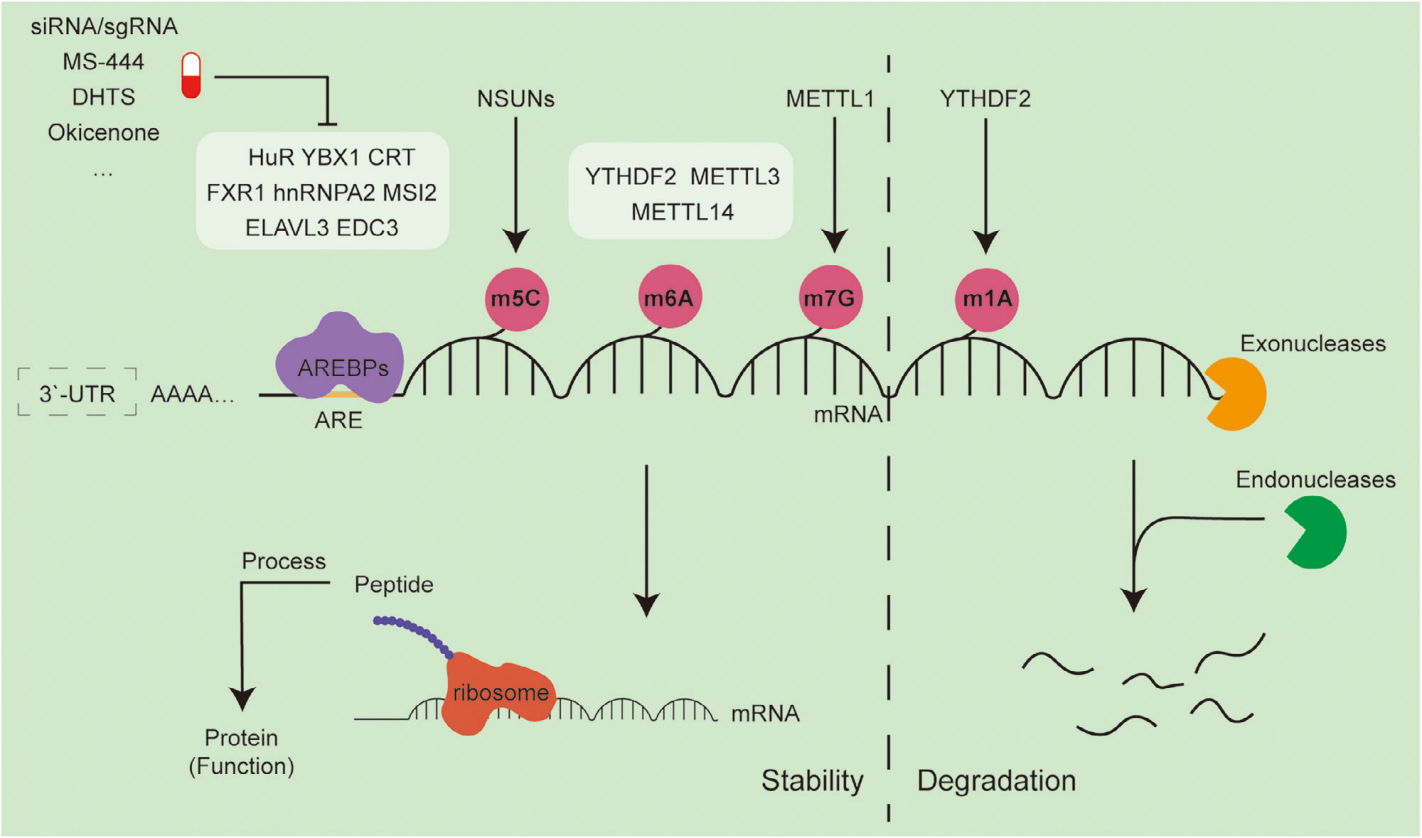
Epitranscriptomic regulation of mRNA stability and degradation in cancer. This schematic depicts the epitranscriptomic modifications that regulate mRNA fate, particularly in the context of cancer biology. Multiple RNA modifications—5‐methylcytosine (m⁵C), N⁶‐methyladenosine (m⁶A), 7‐methylguanosine (m⁷G), and N^1^‐methyladenosine (m^1^A)—are installed by specific writer enzymes such as NSUNs (for m⁵C), METTL3/METTL14 (for m⁶A), and METTL1 (for m⁷G), and are recognized by reader proteins like YTHDF2. These modifications influence mRNA stability and decay through the recruitment of RBPs and interaction with ARE‐binding proteins (AREBPs), including HuR, YBX1, FXR1, and ELAVL3, which bind AU‐rich elements (AREs) in the 3′ untranslated region (3′‐UTR). Stabilized transcripts are processed into peptides via ribosomal translation, whereas mRNAs marked for degradation are directed to exo‐ and endonucleases. Targeting RNA stability using agents such as MS‐444, DHTS, and Okicenone, or siRNA/sgRNA‐based approaches, represents a promising avenue for RNA‐targeted cancer therapies.

mRNA stability is positively correlated with gene expression. In general, the longer the half‐life of a coding mRNA, the greater its stability, and the higher the corresponding gene expression level  [127, 128]. Conversely, mRNA degradation—the final step in mRNA metabolism—is irreversible and directed by cis‐regulatory elements, executed by exonucleases and endonucleases [129]. Faster degradation rates lead to decreased gene expression [128]. To maintain cellular homeostasis, cells tightly regulate the dynamic balance between mRNA stability and degradation through multiple mechanisms, ensuring gene expression remains within a physiological range. Structurally, mature mRNAs are stabilized by a 5′‐cap (m7GpppN) and a 3′‐poly(A) tail, which protect transcripts from nuclease‐mediated degradation and prolong their half‐life [129]. However, this balance can be disrupted during tumorigenesis, as mRNA stability and degradation become aberrantly regulated under oncogenic stress (Figure 2) [130, 131].

RBPs are critical posttranscriptional regulators that modulate mRNA stability and degradation by binding to specific cis‐elements via conserved domains [132]. Classical mRNA decay involves deadenylation, 5′ decapping, and exonucleolytic degradation from either the 5′ or 3′ direction [129]. One of the most well‐studied regulatory elements is the AU‐rich element (ARE), located in the 3′‐untranslated region (3′‐UTR), which promotes poly(A) tail shortening and facilitates mRNA decay [133].

**FIGURE 3:**
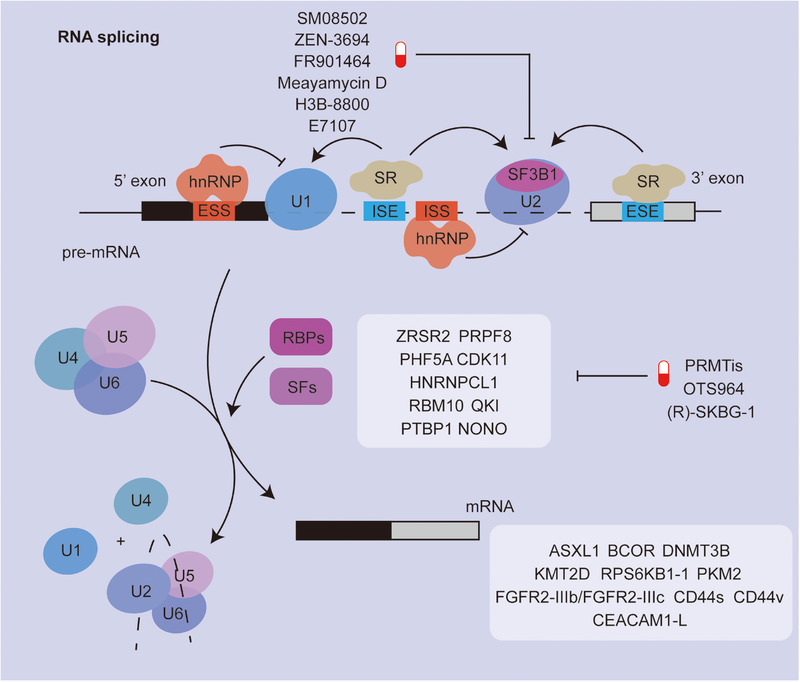
Schematic overview of RNA splicing regulation and its therapeutic targets in cancer. This diagram illustrates the dynamic assembly of the spliceosome on pre‐mRNA, involving key small nuclear ribonucleoproteins (snRNPs: U1, U2, U4, U5, and U6) and splicing factors (SFs), including serine/arginine‐rich (SR) proteins and heterogeneous nuclear ribonucleoproteins (hnRNPs). Regulatory elements such as exonic/intronic splicing enhancers (ESE, ISE) and silencers (ESS, ISS) modulate splice site recognition. Core splicing factor SF3B1, a frequent mutational hotspot in cancer, is highlighted. Cancer‐associated RBPs and splicing factors (SFs) such as ZRSR2, PRPF8, and HNRNPCL1 contribute to oncogenic alternative splicing. Small‐molecule inhibitors targeting splicing machinery (e.g., H3B‐8800, Meayamycin D, E7107) and PRMT inhibitors (e.g., OTS964, (R)‐SKBG‐1) are shown as emerging therapeutic strategies. Aberrant splicing events impact key cancer‐related genes including ASXL1, CD44 variants, FGFR2 isoforms, and CEACAM1‐L, underscoring the pathological relevance of splicing dysregulation in tumorigenesis.

ARE‐binding proteins (ARBPs) recognize these motifs to either destabilize or protect transcripts [134]. Among them, human antigen R (HuR) is a prototypical stabilizing ARBP that enhances the half‐life of ARE‐containing mRNAs across multiple tumor models [135]. HuR has been implicated in several oncogenic processes, including invasion, metastasis, and drug resistance, and its overexpression is consistently associated with poor clinical prognosis. For instance, HuR interacts with phosphorylated UGDH to enhance the stability of SNAI1 mRNA, initiating EMT and promoting cancer cell migration and metastasis [136]. HuR also stabilizes cyclooxygenase‐2 mRNA, correlating with poor outcomes in PCa [137]. In pancreatic cancer, HuR maintains the stability and expression of PIM1, contributing to chemotherapy resistance [138]. Moreover, HuR facilitates the expression of proangiogenic proteins such as VEGF and MMP9, supporting tumor vascularization [139] (Figure 3)

Other ARBPs, including calreticulin  [140], FXR1 [141], hnRNPA2 [142], ELAVL3 [143], MSI2 [144], and YBX1, similarly bind AREs and enhance the mRNA stability of various proto‐oncogenes. In breast cancer cells, YBX1 stabilizes CDK1 mRNA, promoting proliferation [145]. In NSCLC, YBX1 is recruited by RBMPs to the TGFBR1 promoter [146], activating the TGFBR1/SMAD2/3 pathway, thereby sustaining stemness and EMT characteristics, and ultimately inducing radio resistance and cisplatin resistance. Proteins in the P‐body complex, such as EDC3, directly participate in 5′–3′ mRNA degradation [147]. Hyperphosphorylated EDC3 impairs P‐body assembly, reducing mRNA decay and prolonging the half‐life of oncogenic transcripts, thereby driving tumor progression [148].

Epigenetic modifications at the RNA level also influence mRNA stability and degradation. RBPs often act as mediators or “readers” of RNA modifications. Among these, m^6^A is the most abundant internal modification in eukaryotic mRNAs [10]. m^6^A typically functions as a destabilizing mark that shortens transcript half‐life. YTHDF2, a well‐characterized m^6^A reader, contains a C‐terminal YTH domain that recognizes m^6^A‐modified RNAs and an N‐terminal domain that directs them to RNA degradation machinery in the cytoplasm [149]. Under the influence of the m^6^A “writer” METTL3, several tumor suppressor mRNAs become destabilized and undergo rapid decay. In glioblastoma, hnRNPA2B1 mediates m^6^A modification of SREBP2 and LDLR mRNAs, triggering de novo cholesterol biosynthesis and promoting malignant stem‐like phenotypes [150]. Interestingly, hypomethylation at the hnRNPA2B1 promoter also participates in this process. In AML cells, YTHDC1 requires m^6^A for liquid–liquid phase separation to form nuclear YTHDC1–m^6^A condensates, protecting m^6^A–mRNAs from degradation via the PAXT complex and exosome‐associated pathways, thereby maintaining mRNA stability and regulating cancer cell survival and differentiation [151]. Furthermore, ZFP36L2 binds to the 3'‐UTRs of key myeloid maturation genes, promoting their mRNA decay and suppressing terminal differentiation. Genetic inhibition of ZFP36L2 restores the stability of these target transcripts and triggers myeloid differentiation in leukemic cells [152].

In breast cancer, METTL3 and YTHDF2 synergistically promote the degradation of LATS1 mRNA via the Hippo signaling pathway [153], enhancing tumor progression. In bladder cancer, FGF14–AS2 is downregulated in an m^6^A‐dependent manner by YTHDF2, facilitating osteoclastogenesis and bone metastasis [154]. METTL3 and YTHDF2 also cooperatively degrade UBXN1 mRNA, thereby promoting NF‐κB activation and glioma progression [155]. Additional writers and readers, such as METTL14 in hepatocellular carcinoma (HCC), stabilize oncogenic transcripts like SIRT6 [156]. In hematologic malignancies, IGF2BP3 enhances RCC2 mRNA stability, promoting leukemogenesis [157]. In AML, high IGF2BP2 expression is associated with poor prognosis. IGF2BP2 regulates glutamine metabolism in an m^6^A‐dependent manner by stabilizing key targets such as MYC, GPT2, and SLC1A5 [158]. Moreover, KHSRP recognizes and stabilizes FAK pathway mRNAs, including MET, ITGAV, and ITGB1, in an m^6^A‐dependent manner, thereby activating downstream FAK signaling to promote PDAC progression [159].

In contrast, the m^6^A “eraser” FTO removes methylation marks, thereby increasing mRNA stability and altering gene expression. In pancreatic cancer, FTO demethylates NEDD4 mRNA, activating the PTEN–PI3K–AKT pathway and inducing gemcitabine resistance [160]. FTO is highly expressed in relapsed AML, where it downregulates FOXO3 methylation, impairing cell differentiation and maintaining malignant phenotypes [161]. ALKBH5 promotes m^6^A demethylation of FOXO1 mRNA in DOX‐resistant TNBC cells, increasing its stability and enhancing ROS resistance during chemotherapy [162].

Additional RNA modifications also regulate mRNA stability. For example, 7‐methylguanosine (m^7^G) modification catalyzed by METTL1, which is upregulated by the P300/SP1 complex, enhances CDK14 mRNA stability [163], promoting castration‐resistant PCa (CRPC). The NSUN family catalyzes m^5^C methylation on mRNAs such as GRB2 and RNF115, stabilizing them and contributing to esophageal squamous cell carcinoma and poor prognosis in HCC [164, 165], respectively. In bladder cancer, oncogenic transcripts such as PKM2 and BCL2 are stabilized via m^5^C methylation [166, 167]. NSUN4‐mediated m^5^C modification of CDC42 mRNA enhances its binding to BMPREF and modulates stability, thereby driving glioma progression [168]. Furthermore, in breast cancer, methylation of KRT7–AS at A877 enhances the stability of the KRT7–AS/KRT7 mRNA duplex via the IGF2BP1/HuR complex, while YTHDF1/eEF‐1 contributes to FTO‐regulated translation elongation of KRT7 mRNA, promoting lung metastasis [169].

Interestingly, m^1^A modifications may function in opposition to m^6^A. In ovarian and breast cancers, YTHDF2‐mediated removal of m^1^A paradoxically stabilizes CSF1 mRNA and promotes tumor invasion [170]. Methylation marks also influence the tumor immune microenvironment. METTL3 enhances PD‐L1 expression in an m^6^A‐dependent manner, leading to resistance to CD8+ T cell cytotoxicity [171, 172]. Knockdown of METTL3 or METTL14 in tumors increases CD8+ T cell infiltration and the secretion of IFN‐γ, CXCL9, and CXCL10 [173]. Conversely, METTL3 deficiency may delay IRAK‐M mRNA degradation in macrophages, impairing TLR‐mediated activation [174]. These findings highlight the context‐dependent role of RNA methylation in immune modulation and the need to account for cell‐type heterogeneity when designing immunotherapy strategies. Small‐molecule inhibitors targeting the METTL family, such as STM2457, F039‐00027, 460‐0250, and RSM3, have been widely utilized and validated in numerous preclinical studies for their antitumor efficacy, providing conceptual evidence supporting the therapeutic potential of targeting mRNA methylation in cancer treatment [175, 176, 177]. Meanwhile, STC‐15, a novel agent targeting RNA methylation, is currently undergoing a phase I clinical trial to evaluate its safety (NCT05584111). In addition, multiple pharmaceutical companies are actively filing patents for therapeutic strategies based on targeting RNA modifications as a novel mechanism of action [178].

Several therapeutic strategies targeting RNA methylation have been proposed, but here we focus on HuR‐targeted approaches as a model for manipulating mRNA stability in cancer. First, silencing or knocking out the HuR gene using small interfering RNAs (siRNA) or sgRNA significantly inhibits tumor growth in various types of cancers [179]. Delivery systems such as 3DNA nanocarriers or liposomes improve the in vivo half‐life of these agents, with preclinical studies in ovarian, lung, and melanoma models demonstrating efficacy and safety, including prolonged survival and reduced metastasis [180]. In addition, disrupting HuR dimerization impairs its function. Small molecules like MS‐444, 15,16‐dihydrotanshinone‐I (DHTS), and okicenone prevent HuR dimer formation and cytoplasmic translocation, thereby attenuating its stabilizing effect [135]. These compounds reduce tumor growth and enhance chemo‐ and radiosensitivity in preclinical models of tumors [181, 182, 183, 184]. SRI‐42127, a recently developed inhibitor, interferes with HuR dimerization and exhibits potent cytotoxicity in glioma cell lines while significantly suppressing tumor growth in vivo [185]. Finally, interfering with HuR–mRNA binding offers another therapeutic avenue. KH‐3 and DHTS are two known inhibitors that block HuR‐mRNA interaction [186]. In preclinical models of pancreatic, breast, prostate, colorectal, and cervical cancers, these agents enhance the efficacy of chemotherapy [187, 188, 189, 190]. Other compounds, such as CMLD‐2, suramin, AZA‐9, quercetin, b‐40, and b‐41, also disrupt HuR–mRNA binding, although their effects are limited to in vitro studies [135]. These findings underscore the therapeutic potential of targeting mRNA stability, especially in combination with standard therapies. However, further clinical trials are necessary to confirm the safety and efficacy of these approaches in cancer patients.

### 2.3 RNA Splicing Extends the Types of RNA

In eukaryotic cells, precursor messenger RNA (pre‐mRNA) must undergo precise intron removal and exon ligation, regulated by cis‐elements and the spliceosome complex, to generate mature mRNA [191, 192]. AS enables one gene to produce multiple mRNA isoforms, significantly expanding the proteome beyond the ∼20,000 coding genes, contributing to tissue diversity and function [193]. The core spliceosome comprises five small nuclear ribonucleoproteins (snRNPs)—U1, U2, U4, U5, and U6—with numerous auxiliary splicing factors such as RNA‐binding motif proteins (RBMs), serine/arginine‐rich (SR) proteins, and heterogeneous nuclear ribonucleoproteins (hnRNPs) that modulate exon selection [194, 195, 196].

In cancer, aberrant splicing frequently occurs, resulting in tumor‐specific isoforms that promote malignant phenotypes via exon skipping, intron retention, or cryptic splice site usage [15, 197].

Mutations in core spliceosome components are common; SF3B1 mutations are found in ∼30% of MDS, 5% of chronic lymphocytic leukemia (CLL), and subsets of breast, pancreatic, and uveal melanoma [198, 199, 200, 201]. These mutations disrupt branch point recognition, driving oncogenesis via altered transcript repertoires [202, 203]. Similarly, mutations in SRSF2, U2AF1, ZRSR2, and PHF5A affect 3′‐splice site recognition, altering splicing of key oncogenes or tumor suppressors such as ASXL1, DNMT3B, and KMT2D [204, 205, 206, 207, 208]. Dysregulated splicing alters isoforms of TP53, RPS6KB1, PKM, and others, conferring cancer hallmarks such as metabolic reprogramming, EMT, immune evasion, and therapeutic resistance [209].

In a cohort of patients in Ref. [210], TP53 was one of the most frequently altered genes due to intron retention, giving rise to over 12 splice variants linked to poor prognosis and chemoresistance in breast, ovarian cancers, and AML [210]. Mutations in the RPS6KB1 gene create a shorter isoform (RPS6KB1‐1), differentially activating mTORC1 signaling in breast and lung cancers [211]. PKM AS favors the PKM2 isoform, commonly associated with shorter survival and late‐stage tumors [212]. Specific RBPs also regulate cancer‐related splicing. For example, PTBP1 promotes the MEIS2‐L isoform in bladder cancer, enhancing migration, invasion, and lymph node metastasis, while NONO modulates SETMAR splicing to suppress metastasis [213]. NONO facilitates the retention of the second intron within the PRMT1 precursor mRNA, leading to activation of the PRMT1/c‐MYC signaling pathway. In glioblastoma, inhibition of SF3B1 disrupts the mTOR/β‐catenin signaling axis through aberrant BCL2L1 splicing, ultimately influencing patient survival. SF3A2 promotes the expression of the oncogenic splice variant MKRN1‐T1, thereby accelerating TNBC progression. Additionally, ERα RNA binding mediates the AS of XBP1, contributing to tamoxifen resistance formation; this suggests that RNA–RNA interactions are also involved in the splicing regulation process.

AS events are involved in multiple aspects of tumor progression. Imbalance between FGFR2 isoforms IIIb and IIIc triggers EMT in various tumor types [191]. CD44, a CSC marker and EMT regulator, generates multiple isoforms including CD44s and CD44v, both linked to increased tumor proliferation and invasiveness [214]. Recent studies indicate that AS events are associated with perineural invasion (PNI) in peripheral tumors. Specifically, the LAMA3 splice isoform retaining the G45 domain promotes neurite outgrowth. AS also contributes to stromal remodeling and angiogenesis. Splice variants of extracellular matrix proteins such as fibronectin and tenascin‐C affect chemoresistance and tumor progression [215, 216]. VEGFA isoforms differ in their ability to stimulate angiogenesis, and antiangiogenic variants are often downregulated in advanced cancers [217, 218]. Moreover, AS may reshape the tumor immune microenvironment. For example, CEACAM1‐L suppresses receptor signaling, and MYD88s diminishes TLR signaling [219]. FLG–AS1 directly interacts with HNRNPU to regulate the AS of CSF1, thereby promoting M2 polarization of TAMs in PDAC.

Due to the inherent instability of dysregulated splicing, cancer cells are more vulnerable than normal cells to spliceosome disruption, making RNA splicing an attractive therapeutic target. Splicing factor activity is often regulated by posttranslational modifications such as phosphorylation and methylation. Protein arginine methyltransferases (PRMTs) and kinases influence splicing accuracy and represent promising intervention points. PRMT inhibitors have demonstrated efficacy in lymphomas and leukemias, with several trials underway [220]. CDK11, essential for spliceosome activation, is inhibited by OTS964, which reduces CDK11 phosphorylation and promotes chromosomal aberrations, polykaryocytosis, and apoptosis in cancer models [221]. Serine/arginine‐rich protein kinase 1 (SRPK1) inhibitors suppress SR protein phosphorylation, affecting VEGF isoform splicing and xenograft growth [222, 223, 224]. Other kinases such as CLK1, CLK2, and CLK4 also modulate SR protein activity [225]. The first CLK inhibitor, SM08502, recently entered clinical trials and demonstrated antitumor effects by suppressing the Wnt pathway [226]. Although promising, the side effects of kinase inhibitors remain a concern, highlighting the need for higher specificity. Additional therapies include the BET bromodomain inhibitor ZEN‐3694, which, in combination with enzalutamide, shows efficacy in CRPC trials [227]. The HSP90 inhibitor onalespib downregulates over 500 transcripts and reduces AR‐V7 in PCa [228].

Targeting core components of the RNA splicing machinery has emerged as a promising therapeutic strategy, as these proteins play a pivotal role in posttranscriptional RNA processing. Splicing factor 3B subunit 1 (SF3B1) and PHD finger protein 5A (PHF5A), both essential subunits of the U2 snRNP complex, are directly involved in branch point sequence (BPS) recognition and exon–intron boundary definition. In vitro studies have shown that FR901464, a natural product‐derived compound, effectively inhibits SF3B1 and PHF5A, thereby preventing the recognition of the branch point [G] site [229]. This disruption leads to global interference with splicing events and subsequent cytotoxicity in HeLa cells in preclinical models [230]. However, FR901464 has not yet been evaluated in solid tumors. Meayamycin D, a synthetic analogue of FR901464, has demonstrated the ability to modulate AS of the antiapoptotic gene MCL‐1. In recent studies, it exhibited synergistic efficacy with venetoclax in drug‐resistant lung cancer cells [231]. Nonetheless, its therapeutic potential in cancer models or clinical settings remains to be determined through further investigation. Another small molecule, E7107, also targets core splicing factors and underwent evaluation in phase I clinical trials for advanced solid tumors [232]. However, the trial was prematurely terminated due to severe adverse effects, including vision loss in enrolled participants [233]. More recently, H3B‐8800, a selective SF3B1 modulator, has shown a favorable safety profile in a phase I clinical study involving patients with hematological malignancies [234]. While these early results are promising, additional studies are needed to assess its therapeutic efficacy, define optimal dosing regimens, and explore its applicability to solid tumors. Additionally, the NONO inhibitor (R)‐SKBG‐1 has entered early‐phase clinical trials, and its safety and efficacy in cancer patients remain to be further investigated [235].

### 2.4 RNA Transport Endows RNA with Spatial Properties

**FIGURE 4:**
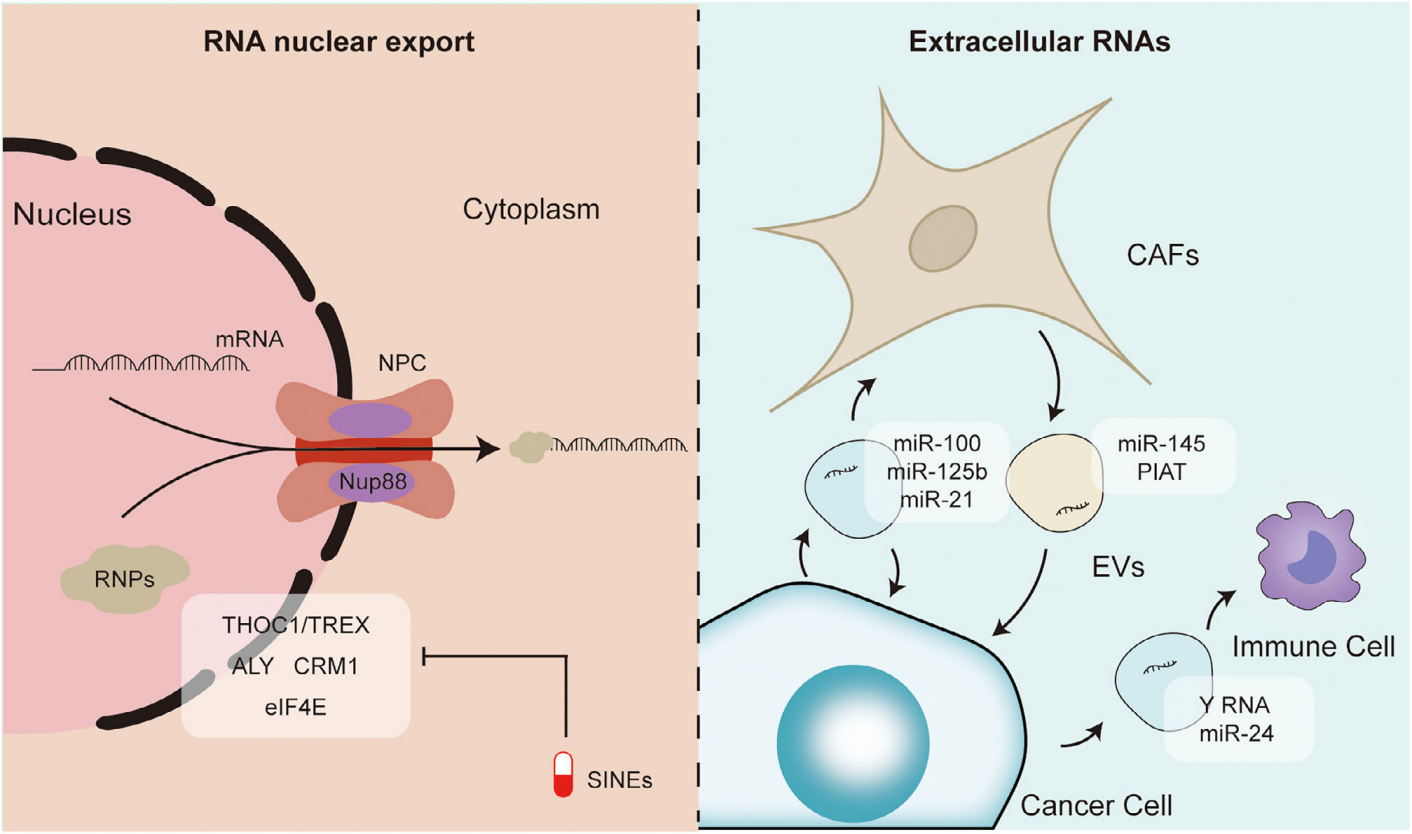
Mechanisms of RNA nuclear export and extracellular RNA‐mediated intercellular communication in cancer. This illustration outlines the two interconnected processes of RNA nuclear export and extracellular RNA (exRNA) trafficking in the tumor microenvironment. (Left) In the nucleus, messenger RNA (mRNA) transcripts are exported to the cytoplasm through the nuclear pore complex (NPC), where nucleoporins such as Nup88 coordinate with RNA‐binding proteins (RNPs) and export factors including THOC1/TREX, ALY, CRM1, and eIF4E. The export process can be selectively inhibited by agents such as selective inhibitors of nuclear export (SINEs). (Right) In the extracellular compartment, cancer cells release exRNAs (e.g., miR‐21, miR‐100, miR‐125b) via extracellular vesicles (EVs), which mediate cell‐to‐cell communication. These vesicles are taken up by stromal components like cancer‐associated fibroblasts (CAFs) and immune cells, influencing tumor progression, immune modulation, and microenvironmental remodeling. Specific exRNAs, such as miR‐145 and PIAT from CAFs or Y RNA and miR‐24 from tumor cells, contribute to the dynamic signaling network that sustains malignancy.

The nuclear envelope, containing nuclear pore complexes (NPCs), separates the nucleus and cytoplasm and mediates selective macromolecule transport [236]. The spatial localization of mRNA plays a critical role in regulating gene expression. Aberrant mRNA export disrupts protein translation and compromises cellular homeostasis. Additionally, various noncoding RNAs are exported into the extracellular space via vesicular transport and exocytosis, facilitating intercellular communication and mediating tumor–microenvironment interactions that sustain malignant progression (Figure 4) [236, 237].

During export, mRNAs associate with adaptor proteins that guide their transit through the NPC. Under physiological conditions, this mechanism ensures efficient clearance of aberrant transcripts, thereby safeguarding transcriptome fidelity. However, in cancer, multiple oncogenic signaling pathways converge on the RNA export machinery, making it a critical mediator of malignancy. Elevated expression of the export factor THOC1 has been reported in breast, lung, ovarian, and CRCs, where it promotes tumor growth and metastasis [238, 239, 240]. Overexpression of ALY has also been observed in oral squamous cell carcinoma [241]. Mutations and dysregulation of classical exportins such as CRM1 (also known as exportin 1) are frequent in B‐cell malignancies, gliomas, cervical, and pancreatic cancers [242, 243, 244]. Furthermore, Nup88, a component of the NPC, is often overexpressed in advanced tumors, highlighting its role in cancer progression [245, 246, 247]. Eukaryotic initiation factors (eIF)4E, primarily recognized for its role in cap‐dependent translation, also mediates the nuclear export of specific transcripts and is strongly associated with poor prognosis in several cancers [248, 249, 250]. Conversely, reduced expression of certain export factors may impair tumor suppressor gene expression.

Extracellular vesicles (EVs) have recently emerged as potent mediators of intercellular signaling. These vesicles carry small biomolecules, including RNA, and facilitate both local and systemic communication [251, 252, 253]. Extracellular RNA (exRNA), reflecting dynamic gene expression, shows promise as a diagnostic, prognostic, and therapeutic biomarker.

In CRC, EVs enriched with miR‐100 and miR‐125b promote EMT [254, 255]. In metastatic breast cancer, miR‐21 is upregulated in EVs and activates the Wnt signaling pathway to drive tumor progression [256]. In CLL, Y RNA carried by tumor‐derived EVs is phagocytosed by macrophages, activating the TLR7 pathway and upregulating PD‐L1, thereby promoting an immunosuppressive microenvironment [257]. Nasopharyngeal carcinoma‐derived EVs contain [missing content] that suppress T cell proliferation and differentiation, facilitating immune evasion [258].

Moreover, EVs from liver, pancreatic, and brain tumors establish pre‐metastatic niches in distant organs. In contrast, certain stromal‐derived EVs may exert antitumor effects. For instance, stromal miR‐145 suppresses tumor formation in PDAC and PCa by inducing apoptosis in recipient cancer cells [259]. In pancreatic cancer, PDPN⁺ cancer‐associated fibroblasts (CAFs) secrete PIAT fragments that promote PNI and metastasis [260].

The complex intra‐ and extracellular RNA trafficking network holds considerable clinical potential. For instance, ribavirin, a classical antiviral agent, competitively binds to the m^7^G cap structure of mRNAs, interfering with exportins and reducing the nuclear export and translation of oncogene transcripts [261, 262, 263]. Ribavirin has demonstrated preclinical and clinical activity in early trials involving patients with acute leukemia, CRPC, and head and neck cancers [264, 265, 266]. Antisense oligonucleotides (ASO) targeting eIF4E have shown limited clinical efficacy to date. Leptomycin B (LMB), a CRM1 inhibitor, exhibited significant toxicity, leading to the early termination of clinical trials [267]. New‐generation selective nuclear export inhibitors (SINEs) have been developed to improve the therapeutic window by reducing off‐target toxicity [243]. Among these, selinexor has been approved by the US FDA for the treatment of relapsed or refractory multiple myeloma, showing durable clinical benefits [243, 268].

Meanwhile, exRNA‐based diagnostics are emerging as a promising tool. Noninvasive detection of circulating exRNA, such as miRNA signatures, is being used to monitor therapeutic responses and differentiate disease stages. For example, the “miRScore” panel, comprising 14 circulating miRNAs, effectively distinguishes localized from metastatic CRPC [269]. Blood‐derived exRNAs also show promise as biomarkers in gliomas [270]. Therapeutic targeting of exRNA is currently focused on engineering or blocking EVs. Selectively inhibiting the secretion of tumor‐associated EVs while preserving normal intercellular communication is a major challenge and an area of active research.

### 2.5 RNA Translation is the Final Step in which Encoding RNA is Ultimately Presented as a Protein

Certain mRNAs are selectively translated depending on cellular context and functional state. Nearly all oncogenic signaling pathways are influenced by aberrant translation in cancer. While prior sections discussed indirect RNA metabolic regulation, here we focus on proteins that directly control translation. The process consists of four major steps: initiation, elongation, termination, and ribosome recycling. Among these, translation initiation—predominantly regulated by eIFs—is widely recognized as the rate‐limiting step [271].

Selective mRNA translation promotes tumor cell growth. Multiple eIF subunits, including eIF2, eIF3, eIF4, eIF5, and eIF6, have been associated with poor prognosis across various cancers [272, 273]. For example, increased eIF2 activation promotes the translation of proliferation‐related transcripts. In CRC, Smad7 activates eIF2α to enhance CDC25 translation and drive S‐phase progression [274]. In fibrosarcoma models, GCN2‐mediated phosphorylation of eIF2 facilitates ATF4 translation, promoting tumorigenicity [275]. eIF4‐dependent mechanisms preferentially translate mRNAs with complex 5′‐UTRs, including key proto‐oncogenes such as MYC, MYB, NOTCH, CDK6, and BCL2 [276, 277]. eIF4A enhances cyclin D1 translation, thereby promoting cell cycle progression and invasiveness [278]. eIF4E enhances the translation of PD‐L1, impairing CD8⁺ T cell cytotoxicity and enabling immune evasion [279]. eIF5B enhances p21 translation, contributing to therapeutic resistance in glioblastoma via NF‐κB signaling [280]. Notably, selective suppression of tumor suppressor gene translation by eIFs has been observed in certain tumor samples, suggesting context‐dependent dysregulation [281, 282, 283].

The regulation of translation is further influenced by RBPs and epitranscriptomic modifications. Musashi‐1 (MSI1) and Musashi‐2 (MSI2) are highly expressed in various cancers [284]. MSI1 competes with eIF4G to bind PABP, thereby suppressing translation of tumor suppressors and maintaining stemness [285]. MSI2 enhances translation of Hoxa9, MYC, and IKZF2, amplifying leukemic phenotypes in MLL‐rearranged leukemias [286]. Hepsin, a type II serine protease often overexpressed in PCa, reduces phosphorylation of eIF2α, thereby inhibiting CDK11p58 activity [287]. eIF2β is implicated in cell cycle regulation and proapoptotic signaling in PCa. HuR binds 3′‐UTRs to repress Caspase‐2L and enhance p53 translation, supporting CRC survival [288]. EBP1 binds the 5′‐UTR of AR mRNA to form a CAG‐containing structure that suppresses AR translation [289, 290]. The Raf/MAPK/ERK pathway also enhances eIF4A1 activity, driving cell cycle gene translation and contributing to tumor growth in cutaneous squamous cell carcinoma [291].

Recent studies suggest m⁶A modifications in 5′‐UTRs promote cap‐independent translation. METTL14, a key component of the methyltransferase complex, facilitates m⁶A deposition on MYB and MYC mRNAs in AML, enhancing their translation and supporting leukemic self‐renewal [292]. METTL3 augments translation through direct interaction with the initiation machinery, promoting growth and invasion in lung cancer [293]. YTHDF1, a major m⁶A reader, recruits eIF3 and facilitates ribosome loading, enhancing the rate‐limiting step of translation [294]. In ovarian cancer, YTHDF1 increases translation of specific eIF3 subunits in an m⁶A‐dependent manner, driving global translation and metastasis [295]. eIF3 itself can act as an m⁶A reader, binding to methylated 5′‐UTRs to selectively boost proto‐oncogene translation. Its subunits eIF3A and eIF3B can bind YTHDF1/3 and upregulate translation of YAP1, promoting proliferation, invasiveness, resistance, and metastasis [293, 296].

Targeting eIFs, especially the eIF4 complex, shows promising antitumor potential. Ribavirin, which competes with eIF4E for the m⁷G cap, has been previously discussed [297, 298, 299]. Other inhibitors such as pateamine A and silvestrol interfere with eIF4A, blocking cap‐dependent translation [300]. Silvestrol selectively inhibits G‐quadruplex‐rich mRNAs, many of which encode proliferative and oncogenic proteins [281]. Compounds like 4EGI‐1 and 4Ei‐1 reduce eIF4E's interaction with eIF4G and eIF4A, destabilize cap‐binding, and induce proteolytic degradation of eIF4E [301]. In lung cancer and lymphoma models, 4Ei‐1 disrupts translation and induces apoptosis, while enhancing chemosensitivity to agents like gemcitabine [302].

The oral eIF4E kinase inhibitor eFT508 is in phase II trials for CRC but failed in CRPC due to lack of efficacy [303]. The ASO LY2275796 has shown tolerability in PCa and lung cancer patients but failed to demonstrate clinical benefit, suggesting complex regulatory mechanisms governing eIF4E activity [304]. Further research is warranted to identify novel targets and optimize therapeutic strategies based on eIF dysregulation.

## 3 Therapeutic Strategies Targeting Noncoding RNAs Epigenetics

More than 90% of genomic DNA is transcribed into ncRNAs that do not encode proteins. Mounting evidence shows that ncRNAs actively shape the RNA epigenetic landscape during tumor progression through diverse epigenetic regulations. ncRNAs are broadly classified as (1) housekeeping ncRNAs, including tRNAs, rRNAs, and snRNAs, and (2) regulatory ncRNAs, such as lncRNAs, miRNAs, piRNAs, and circular RNAs (circRNAs) [305, 306]. Although more structurally stable than protein‐coding RNAs, regulatory ncRNAs remain relatively understudied. Recent studies highlight that despite lacking coding potential, regulatory ncRNAs are essential for cellular function, heterogeneity, and tumor biology.

**FIGURE 5:**
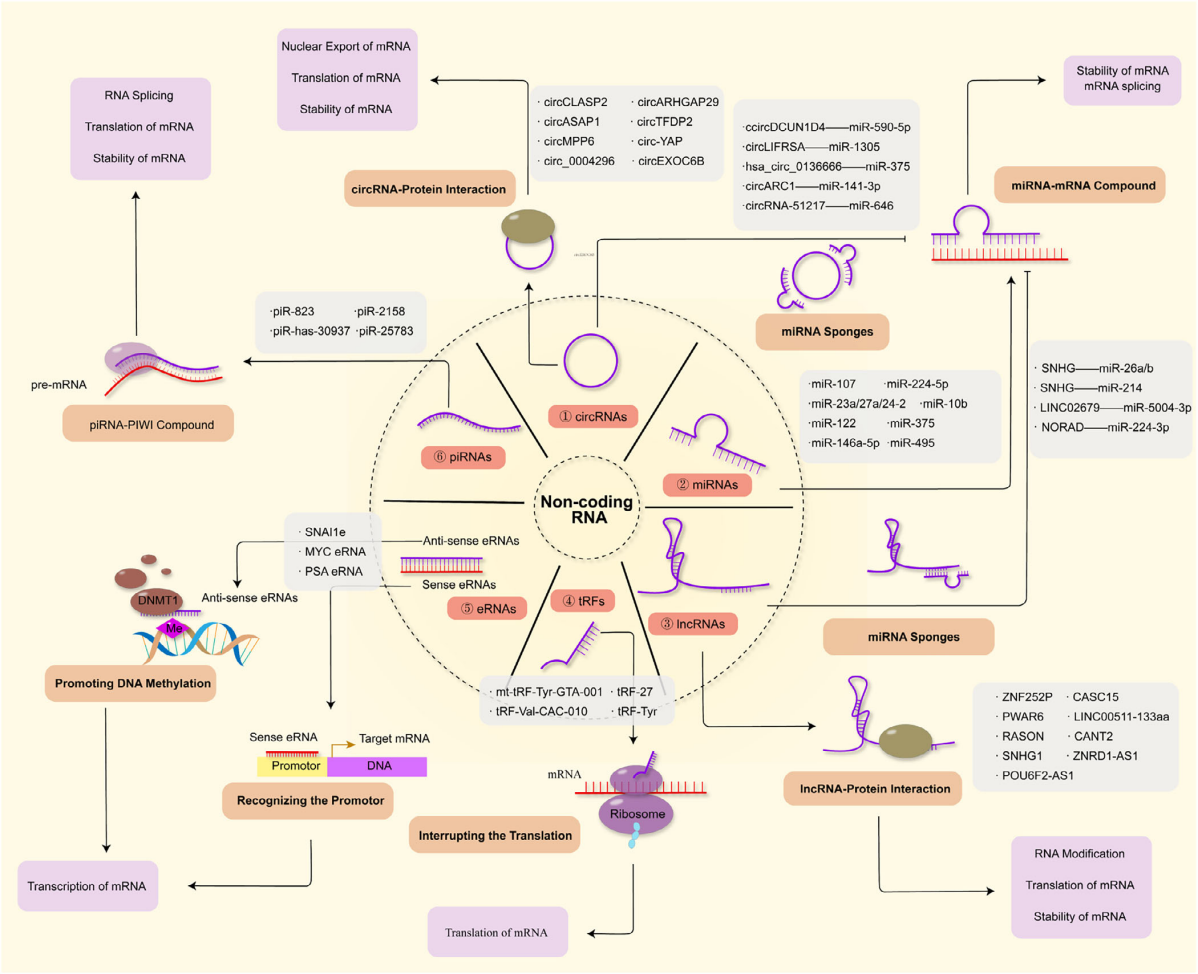
The roles of noncoding RNAs in cancer progression across tumor types. miRNAs regulate mRNA stability and translation through direct base pairing. Both circRNAs and lncRNAs can function as miRNA sponges to inhibit miRNA activity, while also participating in RNA metabolism via interactions with RNA‐binding proteins. piRNAs form complexes with PIWI proteins to regulate mRNA stability, splicing, and translation. Double‐stranded eRNAs are classified as sense or antisense; antisense eRNAs recruit DNMT1 to promote DNA methylation and gene transcription, while sense eRNAs enhance transcription via promoter binding. tRFs have been associated with poor prognosis in multiple cancers, although their molecular mechanisms require further investigation. This figure summarizes representative noncoding RNAs implicated in tumorigenesis across diverse cancer types.

Historically, regulatory ncRNAs were considered by‐products of pre‐mRNA processing and dismissed as “transcriptional noise” [307]. Advances in RNA microarrays and next‐generation sequencing (RNA‐seq) have revealed RNA secondary structure and spatial transcriptomics, highlighting the diverse biological roles of ncRNAs [308]. Due to their high stability and unique functional properties, ncRNAs hold tremendous promise as novel diagnostic biomarkers and therapeutic targets in oncology. In this section, we review recent advances in the understanding of ncRNAs and their roles in tumor progression (Figure 5).

### 3.1 MicroRNAs

MicroRNAs (miRNAs) are small, functional noncoding RNAs of approximately 19–25 nucleotides. They regulate gene expression posttranscriptionally by base‐pairing with complementary sequences on target mRNAs, leading to mRNA degradation or translational repression [309]. A single miRNA can simultaneously target multiple mRNAs, thereby exerting widespread regulatory effects on gene expression. Consequently, miRNAs participate in diverse biological processes, and their dysregulation has been implicated in various human diseases [310].

Recent studies have provided compelling evidence that miRNAs play critical roles in tumor development and progression. Distinct and tumor‐type‐specific miRNA expression patterns have been observed. Functionally, miRNAs may act either as oncogenes (oncomiRs) or tumor suppressors, depending on their molecular targets and biological context. For example, miR‐107 promotes HCC proliferation in vitro and accelerates tumorigenesis in vivo by targeting the mitotic regulator KIF23 [311]. In CRC, miR‐224‐5p confers resistance to 5‐fluorouracil through regulation of S100A4 expression, suggesting its potential as a predictive biomarker for chemoresistance [312]. Overexpression of the miR‐23a/27a/24‐2 cluster has been shown to upregulate PD‐L1 by suppressing CBLB, and to downregulate MHC‐I by targeting MITF, thereby enhancing eIF3B expression and promoting immune evasion as well as resistance to PD‐1/PD‐L1 blockade immunotherapy [313].

In addition, a double‐negative feedback loop has been identified between miR‐122 and the oncogene c‐Myc in HCC. c‐Myc binds to the miR‐122 promoter and represses its expression by downregulating Hnf‐3β, while miR‐122 indirectly inhibits c‐Myc transcription by targeting transcription factors Tfdp2 and E2f1 [313]. In PCa, miR‐375 targets the 3′‐UTR of PTPN4 mRNA, promoting tumor progression and representing a potential therapeutic target. Conversely, miR‐146a‐5p functions as a tumor suppressor in PCa; after androgen deprivation therapy, its downregulation in CAFs reduces EGFR mRNA degradation, thereby weakening its tumor‐suppressive function and facilitating metastasis [314, 315].

Beyond their traditional roles in mRNA regulation, recent evidence shows that miRNAs can also modulate RNA splicing. For instance, miR‐10b interacts with U6 snRNA in glioma cells, leading to AS events that support oncogenesis [316].

The expression and function of miRNAs are also regulated by epigenetic mechanisms. In breast cancer, poly(A)‐specific ribonuclease governs the biogenesis of the 3′‐variant of miR‐125a‐5p [317]. In PCa, FTO demethylase enhances the function of miR‐139‐5p by reversing inhibitory methylation, thus suppressing cancer cell proliferation [318]. Moreover, the m^6^A “writer” METTL3 facilitates the maturation of pri‐miR‐148a‐3p [319] and pre‐miR‐182 [320], thereby regulating pathways such as TXNIP and Wnt/β‐catenin signaling to influence PCa cell proliferation, migration, and invasion. Conversely, miRNAs can also modulate the function of m^6^A‐related enzymes. For instance, miR‐495 downregulates the m⁶A “reader” YTHDF2, disrupting mRNA methylation homeostasis. Its transcription is negatively regulated by KDM5A, which binds to the miR‐495 promoter, highlighting a reciprocal regulatory axis between miRNAs and RNA epigenetic machinery [321].

### 3.2 Long Noncoding RNAs

Unlike miRNAs, long noncoding RNAs (lncRNAs) are defined as transcripts longer than 200 nucleotides that do not encode proteins. LncRNAs exert complex regulatory roles at transcriptional, epigenetic, and posttranscriptional levels, modulating key biological processes such as cell proliferation, migration, differentiation, and apoptosis.

In the nucleus, lncRNAs can interact with transcription factors, splicing regulators, and RBPs to regulate transcription and RNA metabolism processes. In melanoma, CANT2 interacts with PHB2 to regulate transcription of the tumor suppressor gene CCBE1 [322]. In lung cancer, ZNRD1–AS1 exerts its oncogenic effects through the miR‐942/TNS1 axis, regulated by the m^6A reader YTHDC2 [323]. Additionally, m⁶A‐modified lncRNA POU6F2–AS1 reprograms fatty acid metabolism by upregulating FASN, thereby promoting CRC growth [324]. During the maintenance of breast cancer stemness, lncROPM directly binds to the 3'‐UTR of PLA2G16 mRNA, promoting PLA2G16 expression. This reprograms phospholipid metabolism in breast cancer, thereby activating multiple oncogenic pathways associated with stem cell properties. In the cytoplasm, lncRNAs often function as competing endogenous RNAs (ceRNAs), sequestering miRNAs via base pairing and thereby modulating mRNA stability and translation [325, 326]. In CRC, SNHG6 modulates EZH2 expression by sequestering miR‐26a/b and miR‐214 [327]; LINC02679 promotes TRIML2 expression via miR‐5004‐3p in gastric cancer [328]; and in esophageal squamous cell carcinoma, NORAD enhances MTDH expression by sponging miR‐224‐3p [322]. Notably, in CRC, lncRNA SNHG1 exhibits a dual role: within the nucleus, it influences the methylation of cell cycle regulator promoters, while in the cytoplasm, it sequesters miR‐154‐5p, thereby modulating the expression of cell cycle regulators.

Given these diverse functions, lncRNAs have been increasingly implicated in tumor initiation, progression, metastasis, and therapeutic resistance, and are being investigated as potential diagnostic biomarkers and therapeutic targets [329]. For example, ZNF252P has been shown to promote oncogenesis in multiple cancers—including NSCLC, liver hepatocellular carcinoma, colon adenocarcinoma, breast invasive carcinoma, and stomach adenocarcinoma—by facilitating phase separation of HNRNPK and ILF3 and activating c‐Myc at both transcriptional and posttranscriptional levels, forming a feed‐forward regulatory loop [330]. CASC15 promotes gastric cancer peritoneal metastasis via activation of the JNK and p38 pathways [331]. Moreover, CAF‐derived PWAR6 stabilizes NRF2 by competitively binding Keap1, upregulating SLC38A2 expression, enhancing glutamine uptake in CRC cells, and reducing its availability to natural killer (NK) cells, ultimately promoting liver metastasis [332]. In breast cancer, lncSNHG5 is highly expressed in CAFs. It remodels the pre‐metastatic niche via the lncSNHG5–ZNF281–CCL2/CCL5 signaling axis to promote tumor metastasis. Within the pancreatic cancer immune microenvironment, macrophages utilize lncRNA H19 to competitively bind YTHDC1 mRNA, thereby regulating SRSF1‐induced AS of IL‐6 and IL‐10 cytokines.

In addition to their noncoding functions, several lncRNAs have been found to encode bioactive peptides that contribute to tumor development [333]. For instance, LINC00511‐133aa enhances breast cancer stemness and invasiveness via the Wnt/β‐catenin pathway [334], while RASON, encoded by LINC00673, promotes proliferation of PDAC cells by interacting with KRAS [335].

### 3.3 Circular RNAs

CircRNAs represent a distinct subclass of lncRNAs characterized by their covalently closed circular structure, which distinguishes them functionally and structurally from their linear counterparts. CircRNAs are typically derived from pre‐mRNAs through a back‐splicing mechanism, in which a downstream 5′ splice donor joins an upstream 3′ splice acceptor. Based on their genomic origin, circRNAs are classified as exonic, intronic, or exon‐intron circRNAs. Notably, back‐splicing is generally less efficient than canonical linear splicing due to its dependency on specific cis‐elements and trans‐acting factors [336, 337].

In cancer, chromosomal translocations and enhanced mitotic activity frequently contribute to the aberrant generation of specific fusion circRNAs. In addition, dysregulation of splicing factors can promote cancer‐specific circRNA expression. CircRNAs exhibit high species, tissue, and temporal specificity. Their closed‐loop structure confers intrinsic stability, rendering them resistant to exonuclease‐mediated degradation. These properties underscore their functional relevance in cancer progression [338, 339].

Despite their biological potential, circRNAs have long been overlooked due to technical limitations in distinguishing them from linear RNAs. Like other lncRNAs, many circRNAs function as ceRNAs, acting as miRNA sponges to modulate downstream gene expression. Furthermore, they interact with RBPs, influencing RNA metabolism and posttranscriptional regulatory pathways [340].

Recent studies have highlighted the role of circRNAs in tumor progression, particularly in regulating the cell cycle, migration, invasion, and therapeutic resistance. For instance, circDCUN1D4 suppresses HCC progression by sponging miR‐590‐5p and upregulating TIMP3 [341]. In NSCLC, circLIFRSA attenuates malignancy by modulating the miR‐1305/PTEN/AKT axis [342]. In gastric cancer, hsa_circ_0136666 promotes immune evasion via the miR‐375/PRKDC/PD‐L1 axis [343]. In PCa, circARC1 regulates cell invasion through the miR‐141‐3p/MYPT1/p‐MLC2 pathway, while circRNA‐51217 modulates TGFβ1/Smad2/3 signaling by sponging miR‐646 upon AR activation [344].

In addition to their ceRNA functions, some circRNAs modulate RNA–protein interactions. For example, circCLASP2 binds DHX9 to enhance PCMT1 translation in nasopharyngeal carcinoma [345], and circASAP1 induces ferroptosis in renal cell carcinoma via GPX4 regulation through HNRNPC [346]. In CRC, circMPP6 forms a complex with MEX3A to drive UPF1‐mediated PDE5A mRNA degradation, thereby promoting invasiveness [347]. In PCa, circ_0004296 impedes ETS1 nuclear export by retaining EIF4A3 in the nucleus, and circEXOC6B forms a complex with RBMS1 and HuR to stabilize AKAP12 mRNA, suppressing cell migration [348].

CircRNAs are also involved in epitranscriptomic regulation. EIF4A3‐mediated circARHGAP29 modulates c‐MYC mRNA stability via IGF2BP2 binding, contributing to docetaxel resistance in PCa [349]. RBPs such as EIF4A3 promote circRNA biogenesis, as seen in circTFDP2 formation [350]. Additionally, epigenetic modifications—such as m⁶A, m⁷G, and o⁸G—modulate circRNA function. For example, m⁶A‐modified circ‐YAP enhances liver metastasis via the YAP/TEAD axis in CRC, while m⁷G‐modified circKDM1A activates the AKT pathway in CRC [351]. These findings underscore the multifaceted roles of circRNAs in tumor biology and their potential as biomarkers and therapeutic targets.

### 3.4 Other Noncoding RNAs

In addition to the classical ncRNAs discussed above, emerging regulatory noncoding RNAs—including PIWI‐interacting RNAs (piRNAs), enhancer RNAs (eRNAs), and transfer RNA‐derived fragments (tRFs)—have also been implicated in cancer progression and warrant further investigation [352, 353].

*piRNAs* are small ncRNAs of 26–32 nucleotides, initially identified in reproductive system disorders. Functionally analogous to miRNAs, piRNAs associate with PIWI proteins (a subfamily of Argonaute proteins) to suppress transposon activity by silencing transposon RNAs [352, 354]. Growing evidence suggests that piRNAs play pivotal roles in tumor development. For instance, piR‐823 has been demonstrated to regulate proliferation, apoptosis, angiogenesis, and metabolic reprogramming in colorectal, gastric, esophageal, and hematologic malignancies [355, 356, 357, 358]. piR‐2158 represses IL11 expression by competing with FOSL1 for promoter binding, thereby inhibiting breast cancer stemness and angiogenesis [358]. Furthermore, piRNAs also modulate the tumor immune microenvironment. For example, piR‐has‐30937 derived from pancreatic neuroendocrine tumors promotes CD276 expression in macrophages via the PTEN/AKT pathway, facilitating CD276⁺ TAM‐mediated immunosuppression [359]. In ovarian cancer, tumor‐derived exosomal piR‐25783 activates the TGF‐β/SMAD2/3 pathway in fibroblasts, inducing myofibroblast transformation and promoting omental metastasis [360]. In lung adenocarcinoma, piR‐137463 enhances cholesterol biosynthesis through the LOC100128494/miR‐24‐3p/INSIG1 axis, promoting tumor proliferation, migration, invasion, and impairing T cell‐mediated cytotoxicity, while enhancing sensitivity to immune checkpoint blockade [361]. piRNAs also participate in the regulation of RNA modifications. n breast cancer, piR‐651 and PIWIL2 recruit DNMT1 to induce PTEN promoter methylation [362], while piRNA‐823 upregulates DNMT1, DNMT3A, and DNMT3B, resulting in APC gene hypermethylation [363]. Notably, not all piRNAs target transposons, highlighting their broader biological functions beyond transposon regulation.

*eRNAs* are 0.5–5 kb‐long transcripts generated from enhancer regions via RNA polymerase II‐mediated bidirectional transcription. While their mechanisms remain incompletely understood [353, 364], eRNAs are positively correlated with enhancer activity and local mRNA transcription. Antisense eRNAs may recruit DNMT1 to gene bodies, increasing methylation and repressing antisense RNA expression [365], whereas sense eRNAs enhance transcription of nearby genes [366]. In breast cancer, the eRNA SNAI1e interacts with BRD4 to facilitate enhancer‐promoter looping, thereby promoting tumor progression and drug resistance [367]. Another study demonstrated that MYC eRNA cooperates with YEATS2 to recruit the ATAC–HAT complex, thus enhancing MYC transcription in pancreatic cancer [368]. In PCa, PSA eRNA with a TAR‐L motif recruits CYCLIN T1 and activates the CYCLIN T1/P‐TEFb/Pol II Ser2 axis, facilitating castration‐resistant tumor growth [369].

*tRFs* are short ncRNAs (<40 nt) processed from precursor or mature tRNAs, and have recently emerged as critical regulators in cancer biology [353]. tRFs regulate cell proliferation and the cell cycle by modulating the expression of oncogenes or tumor suppressor genes. For example, mitochondrial mt‐tRF–Tyr–GTA‐001 suppresses breast cancer progression by targeting oncogenic transcription factors such as E2F, CCNE1, and FOXM1 [370]. tRF‐27 is associated with tumor size and Ki67 expression in gastric cancer, and serves as a potential prognostic biomarker. In lung adenocarcinoma, tRF–Val–CAC‐010 promotes G2 phase arrest and enhances metastasis [371]. Mechanistically, tRFs often exert their functions via RNA–protein interactions. For instance, tRF‐3019A stabilizes BECN1 by competitively binding STAU1 in colon cancer [372], while tRF–Tyr binds hnRNPD, interfering with its regulation of the c‐Myc 3′‐UTR to inhibit gastric cancer growth [373]. RNA modifications are also involved in tRF biogenesis. In PCa, METTL1 selectively methylates tRNAs at the variable loop, stabilizing them and preventing their cleavage into tRFs [374]. Additionally, pseudouridylation has been implicated in modulating tRF generation [375].

Given their structural diversity, molecular specificity, and regulatory versatility, ncRNAs–including piRNAs, eRNAs, and tRFs–represent promising candidates for therapeutic targeting and biomarker development in PCa. Further elucidation of their roles may provide novel insights into the diagnosis, prognosis, and treatment strategies for this malignancy.

### 3.5 Therapeutic Strategies Targeting RNAs Epigenetics

RNA therapies have emerged as promising strategies in cancer treatment by modulating gene expression through targeting mRNA degradation, translation inhibition, and miRNA‐mediated networks. Current approaches primarily include ncRNA‐related therapies ASOs, siRNAs, short hairpin RNAs (shRNAs), miRNA mimics, antimiRs, engineered circRNAs and coding‐RNA strategies such as mRNA vaccine, CRISRP–Cas9 therapy [376, 377]. ASOs bind complementary sequences on mRNAs to inhibit translation, alter splicing, or trigger degradation via RNase H [377, 378]. Anti‐miRs specifically inhibit oncogenic miRNAs to restore tumor suppressor gene expression [379]. siRNAs and shRNAs induce transcript silencing through the RNA‐induced silencing complex [380], with shRNAs offering improved stability and prolonged effects [381]. miRNA mimics restore tumor‐suppressive miRNA functions by matching or partial matching the sequence of targeted mRNA [382], while circRNAs function as stable miRNA sponges or vectors for therapeutic gene expression [383].

Preclinical studies have demonstrated the potential efficacy of these strategies across various cancers. For example, miR‐26 mimics reverse MYC‐driven suppression to induce tumor regression in HCC [384]; lipid nanoparticles (LNP)‐delivered miR‐34a suppresses CD44⁺ stem‐like cell populations and metastasis in PCa [385]; and intratumoral delivery of the miR‐15a/16 cluster inhibits tumor progression in prostate models [386]. Combinatorial delivery of miR‐34 with miR‐143/145 reduces pancreatic tumor size and induces apoptosis [387]. Moreover, miR‐200c mimics enhance radiosensitivity in lung cancer models [388], and let‐7 family delivery suppresses MYC‐driven tumorigenesis in preclinical studies [389, 390]. Oncogenic miRNA inhibition strategies such as anti‐miR‐10b oligonucleotides have shown efficacy in reducing breast cancer metastasis and inducing glioblastoma regression in mouse models. Concurrently, the FOXP3‐targeting agent AZD8701 was evaluated in preclinical studies using humanized mouse models. Treatment with AZD8701 effectively suppressed regulatory T cell (Treg) function and promoted the antitumor activity of CD8⁺ T cells, as evidenced by alterations in the tumor immune microenvironment. These findings demonstrate the significant immunomodulatory efficacy of AZD8701 [391].

Relevant therapeutic strategies targeting oncogenic drivers have advanced into clinical evaluation. In the earliest clinical trials investigating ASO agents [392], Custirsen (OGX‐011, targeting BCL‐2 mRNA) and imetelstat (targeting telomerase) have entered phase II/III trials for lung cancer and NSCLC, respectively [393, 394]. Oblimersen (G3139, targeting BCL‐2) combined with chemotherapy showed promise in early studies but limited survival benefits in phase III AML trials [395]. Other agents, such as prexigebersen (Grb2 ASO) and siRNAs targeting β‐catenin or PLK1, have demonstrated tumor‐suppressive effects through inhibition of key signaling pathways like ERK, AKT, and Wnt in preclinical models [396]. LNPs and exosomes have significantly improved RNA therapeutic delivery efficiency in vivo. A liposomal formulation of LErafAON (a c‐raf‐targeting ASO), combined with radiotherapy, demonstrated acceptable tolerability in a Phase I clinical trial involving patients with advanced solid malignancies. This supports its potential exploration in future clinical studies [397]. Cobomarsen (MRG‐106), a locked nucleic acid (LNA)‐based miR‐155 inhibitor, demonstrated safety and preliminary efficacy in lymphoma and leukemia clinical trials. Recent clinical studies have validated the safety profile of an ASO targeting IGF‐1R in patients with advanced‐stage HCC, supporting its potential for future efficacy evaluation [398].

CircRNA therapeutics also show potential through miRNA sponging (e.g., targeting miR‐21 in gastric cancer) [399] or encoding tumor suppressor proteins via internal ribosome entry sites (IRES) [400]. Engineered circRNAs may additionally modulate RBP interactions to correct splicing dysregulation in cancer [401].

Despite substantial preclinical progress, clinical translation of ncRNA therapies faces persistent challenges, including delivery efficiency, off‐target effects, immune‐related toxicities, and tumor heterogeneity. For instance, MRX34, a liposomal miR‐34a mimic, was discontinued in multiple clinical trials due to dose‐limiting toxicities. However, recent studies employing preemptive dexamethasone dosing in diverse cohorts of patients with advanced solid tumors demonstrated a manageable toxicity profile and discernible clinical activity. This work collectively provides proof‐of‐concept for miRNA‐based cancer therapeutics [402]. While certain agents (e.g., cobomarsen, STP707) have shown disease stabilization in early trials, large‐scale, robust clinical evidence remains limited.

Furthermore, mRNA vaccines encoding tumor antigens or immune modulators represent a novel immunotherapeutic strategy. LNP‐formulated mRNA vaccines targeting melanoma antigens (gp100, TRP2) or delivering IL‐36γ/IL‐23/OX40L reshape the TME, enhancing immune infiltration and checkpoint response [403]. Clinical‐stage vaccines such as mRNA‐4157 (V940, Moderna) [404] and Autogene cevumeran (BNT122) have demonstrated encouraging efficacy in melanoma and pancreatic cancer, respectively, by eliciting durable neoantigen‐specific T cell responses [405]. Autogene cevumandine, a personalized neoantigen‐specific immunotherapy utilizing uridine‐modified mRNA encapsulated in LNPs has garnered significant attention in recent years. This approach designs multiple neoantigens based on patient‐specific somatic mutation data derived from tumor tissue to stimulate T‐cell immune responses. In this ongoing Phase I study evaluating autologous cevumeran monotherapy (*n* = 30) and combination therapy with atezolizumab (*n* = 183) in patients with advanced solid tumors, objective responses were detected within 23 months of treatment initiation, including one patient receiving monotherapy and two receiving combination therapy, demonstrating promising therapeutic activity [406].

The CRISPR system, derived from a prokaryotic adaptive immune system, has been extensively optimized for precise gene editing in mammalian cells. CRISPR‐based strategies for cancer therapy frequently target the editing of oncogenes and tumor suppressor genes within tumor cells themselves, including disruption of gain‐of‐function oncogenic mutations and restoration of inactivated tumor suppressors [376]. More commonly, however, CRISPR is applied to engineer immune cells, enabling the development of immunotherapies such as CAR‐T cells. Numerous CAR‐T therapies have advanced into Phase I clinical trials across diverse malignancies in recent years. IL‐13Rα2‐targeted CAR‐T cells achieved disease stabilization or better in 50% (29 out of 58) of glioblastoma patients [407]; ROR1‐specific CAR‐T cells demonstrated responses in 2/3 of patients with hematologic or aggressive epithelial malignancies but showed minimal efficacy in immunologically cold tumors [408]. CD19/GCC‐targeted CAR‐T cells yielded a 40% objective response rate in a metastatic CRC cohort and extended median overall survival to 22.8 months [408]. All therapies exhibited favorable tolerability profiles within their respective patient cohorts. Moreover, CRISPR‐based systems can specifically target aberrant RNA modifications for precise regulation, thereby offering novel strategies for cancer therapy [409]. For example, the TRM platform, engineered based on the function of Cas13, has been developed to target METTL3 and METTL14, demonstrating promising therapeutic efficacy, although inevitable off‐target effects have also been observed [410]. In contrast, another tool, TRADES, targets ALKBH5 or FTO and exhibits stronger editing efficiency and higher specificity; however, its large molecular size poses challenges for delivery [411].

Future directions should emphasize combinatorial regimens (e.g., RNA therapeutics with checkpoint inhibitors), personalized strategies (neoantigen‐based vaccines), and improvements in delivery platforms (engineered LNPs, exosomes, circRNAs). Advances in synthetic biology and immune‐oncology will be crucial to bridging the gap from bench to bedside.

## 4 Major Advantages and Future Suggestions of Therapeutic Strategies Targeting RNA Epigenetic

As discussed above, RNA epigenetics‐based therapies (Table 1) capitalize on aberrant RNA metabolic processes occurring during tumor progression, offering promising avenues for effective and personalized cancer treatment. Compared with traditional therapeutic modalities, RNA therapeutics present several notable advantages:

1. Most RNA therapies leverage the principle of Watson–Crick base pairing, allowing for highly specific and relatively straightforward sequence design and synthesis due to the availability of precise RNA sequence information;
2. Unlike DNA‐based therapies, RNA therapeutics do not integrate into the host genome, thereby preserving genomic integrity;
3. Owing to the inherently low immunogenicity of RNA molecules, these therapies are less likely to provoke strong host immune responses;
4. A single RNA‐based therapeutic can simultaneously regulate multiple target genes with high specificity and efficiency;
5. While protein‐based therapies offer similar benefits, RNA therapeutics target a broader array of molecules, as approximately 70% of the human genome is transcribed into ncRNAs, whereas protein‐coding genes comprise a much smaller proportion [412, 413].

**TABLE 1:** The clinical studies and mechanism of action of therapies based on RNA epigenetics.

Disease	Drugs	Targeted gene or mRNA	Mechanism of action	Molecular/phenotypic effect	Phase	NCT
Breast cancer	Custirsen (OGX‐011)	HSP90	ASO	Specifically inhibits the expression of HSP90β through antisense RNA technology disrupts the protein homeostasis network of tumor cells, induces apoptosis, and overcomes drug resistance.	II I	NCT00258375 NCT00471432
	Imetelstat (GRN163L)	hTR	ASO	Inhibits telomerase activity, cuts off the “molecular switch” for infinite proliferation of tumor cells, induces their senescence or apoptosis, and can produce synergistic effects with other antitumor therapies	II I	NCT01256762 NCT00732056
	IVAC‐MUTANOME	Tumor‐associated antigen	Vaccines	On‐demand RNA manufacturing for use in single patients to target multiple neo‐antigens derived from mutated epitopes	I	NCT02316457
	Ribavirin	/	Antiviral drug	Competitively binds the 7‐methylguanosine (m⁷G) cap structure of mRNAs, interfering with exportins, and reducing the nuclear export and translation of oncogene transcripts	I/II	NCT01056757
	Quercetin	HuR	Natural product	Interferes with the interaction between HuR and mRNA, thereby compromising the stability of oncogenic transcripts	I	NCT05680662
	KPT‐330	XPO1	SINE	Specifically targets the nuclear export receptor XPO1, resulting in the nuclear retention of tumor suppressor proteins. This nuclear sequestration enhances their transcriptional regulatory activity, thereby inducing cell cycle arrest and promoting apoptosis.	I/II	NCT05035745
	Onalespib	HSP90	HSP90 inhibitor	By binding to the N‐terminal ATP‐binding pocket of HSP90, it prevents ATP binding and hydrolysis, thereby disrupting multiple oncogenic signaling pathways, inducing tumor cell apoptosis and cell cycle arrest, and reversing drug resistance.	I	NCT02898207
	ZEN003694	BRD2, BRD3, BRD4	BRDi	Inhibiting the binding of BET family members (including BRD2, BRD3, BRD4, and BRDT) to acetylated lysine residues on chromatin modulates the transcriptional activation of various oncogenes.	II	NCT03901469
Liver cancer	TKM‐080301	PLK1	siRNA	Downregulation of PLK1‐induced apoptosis and showed antitumor efficacy	I I/II	NCT01437007 NCT02191878
	ALN‐VSP	VEGF KSP	siRNA	Targeting VEGF and kinesin spindle protein (KSP). Antitumor activity	/	/
	DCR‐MYC	MYC	siRNA	Targets the oncogene MYC Prevents tumor progression	Ib/II	NCT02314052
	RO7070179	HIF1A	ASO	HIF1A inhibition by a decrease of HIF1A mRNA after intravenous (IV) infusion; Exert antitumor effects	I	NCT02564614
	AEG35156	XIAP	ASO	Binds to XIAP mRNA, promoting its degradation or blocking translation, thereby reducing XIAP protein expression. This alleviates the inhibition of apoptosis and induces tumor cell apoptosis to exert antitumor effects.	I/II	NCT00882869
Prostate cancer	Custirsen (OGX‐011)	HSP90	ASO	Specifically inhibiting the expression of HSP90β through antisense RNA technology disrupts the protein homeostasis network of tumor cells, induces apoptosis, and overcomes drug resistance	II II	NCT00327340 NCT00138918
	Quercetin	HuR	Natural product	Interferes with the interaction between HuR and mRNA, thereby compromising the stability of oncogenic transcripts	I/II	NCT01538316
	SM08502	CLK	CLKi	By modulating CLK kinase activity, the regulation of SR proteins is altered, ultimately affecting the WNT signaling pathway and inhibiting tumor progression.	I	NCT05084859
	ISIS EIF4E Rx	eIF4E	ASO	By being fully complementary to eIF4E mRNA, the antisense oligonucleotide induces its degradation and thereby indirectly inhibits the translation of multiple oncogenic proteins.	I/II	NCT01675128
	eFT‐508	eIF4E	/	Inhibiting MNK1/2‐mediated phosphorylation of eIF4E at Ser209, thereby attenuating the translation of oncogenic mRNAs such as MYC and MCL‐1, ultimately suppressing tumor growth	II	NCT03690141
	ZEN003694	BRD2, BRD3, BRD4	BRDi	Inhibiting the binding of BET family members (including BRD2, BRD3, BRD4, and BRDT) to acetylated lysine residues on chromatin modulates the transcriptional activation of various oncogenes.	II	NCT04471974
Lung cancer	TargomiRs	miR‐15 miR‐16	Counteract the loss of the miR‐15 and miR‐16 family miRNAs	TargomiRs are minicells (EnGeneIC Dream Vectors) loaded with miR‐16‐based mimic microRNA (miRNA) and targeted to EGFR that are designed to counteract the loss of the miR‐15 and miR‐16 family miRNAs; Inhibit tumor growth.	I	NCT06615752
	Onalespib	HSP90	HSP90 inhibitor	By binding to the N‐terminal ATP‐binding pocket of HSP90, it prevents ATP binding and hydrolysis, thereby disrupting multiple oncogenic signaling pathways, inducing tumor cell apoptosis and cell cycle arrest, and reversing drug resistance.	I/II	NCT02535338
	ISIS EIF4E Rx	eIF4E	ASO	By being fully complementary to eIF4E mRNA, the antisense oligonucleotide induces its degradation and thereby indirectly inhibits the translation of multiple oncogenic proteins.	I/II	NCT01234038
Pancreatic cancer	Custirsen (OGX‐011)	HSP90	ASO	Specifically inhibiting the expression of HSP90β through antisense RNA technology disrupts the protein homeostasis network of tumor cells, induces apoptosis, and overcomes drug resistance	I I/II	NCT00471432 NCT00138658
	Imetelstat (GRN163L)	hTR	ASO	Inhibits telomerase activity, cuts off the “molecular switch” for infinite proliferation of tumor cells, induces their senescence or apoptosis, and can produce synergistic effects with other antitumor therapies	I II	NCT00510445 NCT01137968
	siG12D LODER	KRAS	siRNA	Achieves precise targeting of KRAS G12D‐mutated tumors through mutation‐specific RNA interference and local sustained‐release delivery, inhibiting tumor progression from multiple dimensions including proliferation, apoptosis, angiogenesis, and the immune microenvironment	I II	NCT01188785 NCT01676259
	Atu027	PKN3	siRNA	Silences the expression of protein kinase N3 (PKN3) in the vascular endothelium; Inhibits tumor growth and reduced tumor metastases in the lymph nodes	I/II	NCT01808638
Ovarian cancer	EphA2 siRNA	EphA2	siRNA	Specifically silences the EphA2 gene through RNA interference, inhibiting tumor progression from multiple dimensions including proliferation, apoptosis, metastasis, angiogenesis, and the immune microenvironment, and can enhance the efficacy in combination with chemotherapy, immunotherapy, and other treatments	/	/
Melanoma	Lipo‐MERIT	NY‐ESO‐1 tyrosinase MAGE‐A3 TPTE	Vaccines	Targets four nonmutated, tumor‐associated antigens that are prevalent in melanoma. Clinical responses are accompanied by the induction of strong CD4+ and CD8+T cell immunity	I	NCT02410733
	RBL001/RBL002	Melanoma‐associated target antigens	Vaccines	Elicits strong antitumor immunity and increases progression‐free survival in patients with metastatic melanoma	I	NCT01684241
Hematologic malignancies	BP1001	Grb2	ASO	Inhibits protein expression, blocks tumor cell proliferation signaling, induces apoptosis, and suppresses angiogenesis	I	NCT01159028
	KPT‐330	XPO1	SINE	Specifically targets the nuclear export receptor XPO1, resulting in the nuclear retention of tumor suppressor proteins. This nuclear sequestration enhances their transcriptional regulatory activity, thereby inducing cell cycle arrest and promoting apoptosis.	I	NCT01607892
	eFT‐508	eIF4E	/	Inhibiting MNK1/2‐mediated phosphorylation of eIF4E at Ser209, thereby attenuating the translation of oncogenic mRNAs such as MYC and MCL‐1, ultimately suppressing tumor growth	I/II	NCT02937675
	Ribavirin	/	Antiviral drug	Competitively binds the 7‐methylguanosine (m⁷G) cap structure of mRNAs, interfering with exportins and reducing the nuclear export and translation of oncogene transcripts	IV II	NCT00500578 NCT00559091
Glioblastoma	NU‐0129	Bcl2L12	siRNA	Stop cancer cells from growing	I	NCT03020017

To further clarify the conceptual framework of RNA‐based therapies, we categorize them into two major strategic approaches: direct and indirect interventions. Direct RNA therapeutics include mRNA vaccines and synthetic ncRNAs [413, 414]. mRNA vaccines facilitate the intracellular synthesis of target proteins or antigens, thereby activating downstream signaling pathways or triggering immune responses [11]. In contrast, artificial ncRNAs achieve gene silencing by binding to complementary RNA sequences, thus downregulating gene expression [413]. Indirect RNA therapeutics involve targeting upstream regulatory mechanisms of RNA epigenetics—such as RNA splicing factors, RNA‐modifying enzymes, or biogenesis regulators—with the goal of pharmacologically modulating RNA fate and function [415, 416].

Given the advantages described above, therapeutic strategies targeting RNA epigenetics hold substantial promise in cancer treatment and are expected to serve as a cornerstone of personalized and precision oncology. Nevertheless, several significant challenges remain that must be addressed before full clinical translation can be achieved. We highlight the following key obstacles and recommendations for future research:

1. *Toxicity from broad RNA epigenetic modulation*:
  RNA epigenetic mechanisms are essential for normal cellular homeostasis and are dysregulated in cancer. Thus, indiscriminate targeting of RNA epigenetics may lead to systemic toxicity. Future studies should clarify the context‐specific roles of RNA modifications in tumor progression to identify more selective and therapeutically viable targets.
2. *Limited validated targets for mRNA vaccines*:
  Despite the clinical potential of mRNA vaccines in oncology, validated tumor‐specific mRNA targets are still scarce. Advances in genome editing (e.g., CRISPR–Cas systems), high‐throughput RNA‐Seq, and systems biology platforms will play a key role in uncovering novel, functionally relevant RNA targets for therapeutic development.
3. *Immune responses and RNA antigenicity*:
  Although RNA is generally considered less immunogenic than proteins, RNA‐based therapies can still trigger significant host immune responses. Strategies to minimize RNA antigenicity—such as RNA chemical modifications and improved delivery vectors—are urgently needed. Third‐generation RNA modifications, including LNAs, peptide nucleic acids, and phosphorodiamidate morpholino oligomers (PMOs), have shown promise in reducing toxicity and immunogenicity. Furthermore, RNA delivery platforms based on nanocarriers are under active investigation and have demonstrated the potential to modulate immune activation while enhancing therapeutic efficacy.
4. *Clinical translation remains limited*:
  Despite promising preclinical findings, many RNA‐based therapies have not yet progressed to clinical application. Additional translational and clinical research is essential to validate the efficacy, safety, and feasibility of targeting RNA epigenetic regulation in cancer therapy.

In summary, RNA therapeutics represent a transformative approach to cancer treatment. Overcoming current translational barriers will be key to unlocking their full clinical potential.

## 5 Conclusion

Epigenetic mechanisms have been extensively studied in the context of tumor progression. However, traditional epigenetic frameworks do not encompass all regulatory factors affecting gene expression during transcription and translation. This review focuses on RNA epigenetics in tumors, examining how DNA and histone modifications influence RNA transcription, and analyzing posttranscriptional processes such as RNA stability, splicing, translation, and localization to provide a comprehensive overview of gene expression alterations during tumor progression.

Recent studies have elucidated various aspects of RNA epigenetics; yet, these processes have not been fully integrated into a cohesive framework. Advances in NGS technologies have enriched gene expression profiling, enabling more detailed and multidimensional characterization of RNA epigenetic processes. By synthesizing existing literature, we have linked various RNA metabolic processes in tumor cells to phenotypes such as proliferation, invasion, metastasis, and drug resistance, while also considering alterations in immune and stromal cells within the immunosuppressive TME. This comprehensive approach facilitates a broader understanding of the interaction networks within the TME. Nevertheless, RNA epigenetics in immune cells remains underexplored, and the mechanisms driving phenotypic variations in immune and stromal cell populations are not yet fully understood. The advent of single‐cell sequencing and spatial transcriptomics technologies has enabled more precise analyses of gene expression changes within immune and stromal cell populations, paving the way for the development of analytical methods and therapeutic strategies targeting RNA epigenetics in tumor, immune, and stromal cells.

Clinical trials have shown that therapeutic strategies targeting RNA epigenetics exhibit significant efficacy in hematologic malignancies, with several agents approved by the US FDA for clinical use. However, the complexity of gene expression and intratumoral heterogeneity in solid tumors may contribute to the limited safety and efficacy of RNA epigenetics‐targeted therapies observed in these patients. Nonetheless, preclinical and clinical studies have indicated that RNA epigenetics‐targeting drugs can modulate the tumor immune microenvironment, suggesting potential in overcoming resistance to immunotherapy; thus, combination therapies may offer a viable approach. Furthermore, off‐target effects leading to dysfunction of key RNA epigenetics‐related enzymes underscore the need for developing highly specific and precise therapeutic strategies. The design of appropriate delivery vectors, identification of specific targets, and implementation of novel technologies are critical for successful clinical translation.

Integrating RNA epigenetics and posttranscriptional modifications into the framework of classical epigenetics during tumor progression highlights the structural and functional diversity of RNA, providing a molecular basis for various pathological changes in tumors. RNA‐based therapeutic strategies represent a highly promising approach. Addressing current off‐target effects and enhancing efficacy through multifaceted approaches will significantly advance clinical translation. In summary, continued investigation into RNA epigenetic processes remains a critical area of tumor research.

## Author Contributions

Writing—original draft: Shanhe Huang, Zonglin Li, Weilong Lin, Ruihui Xie, and Hai Huang. Visualization: Shanhe Huang, Zonglin Li, and Weilong Lin. Project administration: Ruihui Xie and Hai Huang. Review and editing: Ruihui Xie and Hai Huang. All authors read and approved the final manuscript.

## Ethics Statement

The authors have nothing to report.

## Conflicts of Interest

The authors declare no conflicts of interest.

## Data Availability

The authors have nothing to report.
